# The Berlin Brain-Computer Interface: Progress Beyond Communication and Control

**DOI:** 10.3389/fnins.2016.00530

**Published:** 2016-11-21

**Authors:** Benjamin Blankertz, Laura Acqualagna, Sven Dähne, Stefan Haufe, Matthias Schultze-Kraft, Irene Sturm, Marija Ušćumlic, Markus A. Wenzel, Gabriel Curio, Klaus-Robert Müller

**Affiliations:** ^1^Neurotechnology Group, Technische Universität BerlinBerlin, Germany; ^2^Bernstein Focus: NeurotechnologyBerlin, Germany; ^3^Machine Learning Group, Technische Universität BerlinBerlin, Germany; ^4^Neurophysics Group, Department of Neurology, Campus Benjamin Franklin, Charité - University Medicine BerlinBerlin, Germany; ^5^Department of Brain and Cognitive Engineering, Korea UniversitySeoul, South Korea

**Keywords:** Brain-Computer Interfacing (BCI), electroencephalography (EEG), covert user states, machine learning, mental workload, video quality, implicit information, cognitive neuroscience

## Abstract

The combined effect of fundamental results about neurocognitive processes and advancements in decoding mental states from ongoing brain signals has brought forth a whole range of potential neurotechnological applications. In this article, we review our developments in this area and put them into perspective. These examples cover a wide range of maturity levels with respect to their applicability. While we assume we are still a long way away from integrating Brain-Computer Interface (BCI) technology in general interaction with computers, or from implementing neurotechnological measures in safety-critical workplaces, results have already now been obtained involving a BCI as research tool. In this article, we discuss the reasons why, in some of the prospective application domains, considerable effort is still required to make the systems ready to deal with the full complexity of the real world.

## 1. Introduction

Since the discovery of electrical brain activity and the invention of the Electroencephalogram (EEG), by Hans Berger in 1924 (Berger, 1929), there have been many ideas and dreams about how to exploit this access to the center of human thoughts and emotions and the control of actions. Gray Walter developed the toposcope in 1951 which visualized rhythmic brain activity in 22 spatially laid out cathode ray tubes, each of which showing amplitude and phase in spiral displays (Walter and Shipton, 1951; Bladin, 2006). In the 1960s, realtime EEG was used in artistic performances (e.g., Lucier, 1965; Straebel and Thoben, 2014) and for neurofeedback training (Kamiya, 1969). The latter laid the foundation for possible clinical applications by neurofeedback (Sterman and Friar, 1972). This research led to the idea that human intentions could be transmitted directly from brain to computer (Vidal, 1973). By voluntarily acquiring certain mental states, the user of such a Brain-Computer Interface (BCI) could communicate or control a technical device while circumventing the need for any muscular activity (Dornhege et al., 2007; Wolpaw and Wolpaw, 2012). Clinical application has been the principal goal of BCI research for about four decades (Elbert et al., 1980; Kübler et al., 2005; Shih et al., 2012; Faller et al., 2014; Gallegos-Ayala et al., 2014; Hill et al., 2014; Morone et al., 2015; Soekadar et al., 2015).

In the last decade, the potential of *non*-medical applications of BCI technology has increasingly drawn renewed attention (Müller et al., 2008; Blankertz et al., 2010; van Erp et al., 2012). Two of the five application scenarios defined in the roadmap of brain/neural-computer interaction (BNCI Horizon 2020, 2015; Brunner et al., 2015), primarily target non-medical areas. The *enhance* scenario comprises applications that enhance human functions or user interactivity by adapting the device to their momentary mental state, for example, and by exploiting implicit information about the user's intention. The *research tool* scenario utilizes real-time analysis of neural signals to investigate and understand brain and cognitive functions.

In this review, we deliberately focus on certain developments that we discuss in some detail. We hope that this focused view on our own work will nevertheless be of use to the interested reader and still provide a broad account of the developments of BCI-enhanced neurotechnology. We leave aside a wide range of other relevant research, though it is no less important, pointing here exemplarily just at a few: One potentially interesting aspect for enhancing human-computer interaction is the possibility of predicting the subject confidence of participants (Graziano et al., 2015). Research on memory encoding processes is converging toward the feasibility of predicting the success of memorization by observing brain signals before and/or during encoding (Noh et al., 2014; Cohen et al., 2015). An elaboration of these techniques toward online prediction based on single-trial data would have interesting applications in adaptive learning software. A collective computer game was used to collect data sets from 523 participants in a single night who controlled an immersive art environment with the mental states of relaxation and concentration (Kovacevic et al., 2015).

## 2. Overview of the applications

Since our first review (Blankertz et al., 2010), 6 years have passed, bringing a wealth of novel developments. In this article, we review a selection of our research in this direction. Unlike in most medical applications, these approaches do not employ BCIs to let the user consciously transmit information to the computer. Instead, they use BCIs to infer covert user states and implicit information.

### 2.1. Types and components of brain signals being exploited

Brain signals are either of the *exogenous* type, in which corresponding processes are elicited by external stimuli, or they are *endogenous*, originating from the participant independently of external events. Examples of endogenous brain signals include preparatory signals, such as the readiness potential (RP) and ongoing oscillations, like the alpha rhythm of the visual cortex. The exogenous signals that originate from the cortex can be roughly divided into those that are as being perception related, e.g., the visual evoked potential (VEP), the N1–P2 complex, and the steady-state visual evoked potential (SSVEP) or as being related to cognitive processes such as the P300 or the late positive component. The BCI applications that are reviewed here exploit all of these different types of brain signals—sometimes they are used in combination, sometimes just one alone.

### 2.2. Stratification of use cases

#### 2.2.1. Type I: BCI as a tool for research

BCIs can provide an instantaneous estimate of the mental state and processing of an experimental subject. This possibility offers novel opportunities for (notably non-BCI-related) research. In Section 7, we show how closed-loop technology can be harnessed to study the relationship between preparatory signals (the RP) and corresponding actions—particularly the vetoing of such actions—and how it can thereby contribute to a fundamental question in cognitive neuroscience. The studies reviewed in Section 8 similarly target research questions in a different domain: the cognition and processing of music. In contrast to previous studies, it is not the real-time aspect that is exploited here, but rather the increased sensitivity to the methods that have been developed in BCI contexts. These algorithms enable a step forward in the analysis of music perception from fundamental studies with artificial and repeated stimuli toward investigating more natural behavior in music listening.

#### 2.2.2. Type II: BCI as a tool to improve devices, interfaces, or infrastructure

By providing brain-based measures while a participant is using a certain interface or product, or is interacting in a certain environment, one can assess and compare different variants and settings. A navigation system for a car may be tested and optimized with respect to how little it distracts the driver from the driving task as quantified by neural measures of workload or focused attention (Kohlmorgen et al., 2007; Blankertz et al., 2010). This is an example of an effect about which it is difficult to obtain reliable and unbiased measures through means such as questionnaires. The quantification of mental workload under real world conditions, however, is still a challenge for neurotechnologies. This ability would open an attractive range of applications. Section 5 presents some novel data analysis techniques that can be useful in this context. A workload index could also be employed to assess safety-critical aspects of infrastructure, such as harbors and bridges that require demanding maneuvers (Miklody et al., 2016).

An example of this application type in a quite different domain is given in Section 4. Visual perception of slight distortions in videos is probed and quantified as SSVEP amplitude, giving rise to a potential means of assessing the quality of video codecs. We contrast this approach with alternatives that employ cognitive event-related potential (ERP) components.

#### 2.2.3. Type III: BCI as a device to enhance or facilitate human actions

The direct control of computer applications with a BCI does not seem to be a realistic objective for healthy users. Yet some mileage could well be gained from using a BCI to obtain implicit information from a user during computer use. Added to the explicit information the computer obtains during an interaction, such implicit information should enable computers to understand human users better. We summarize studies dealing with various aspects of such implicit interactions in Section 6.

Section 3 explores the potential of BCI technology to provide information about an intended action before its execution. This potential is exemplified in the prediction of emergency braking when driving. A similar methodology is used in Section 7 as described above. But whereas the allter[PS1] use case exploits endogenous signals only, the prediction of emergency braking is based on a combination of different components, including perceptual and cognitive exogenous signals. For potential applications of the possibility of speeding up actions with BCIs, however, one needs to consider that this by-pass bears the risk of triggering actions on premature intentions and that it eliminates the possibility of vetoing the respective action in the last moment. This aspect is backed by the results reviewed in Section 7. The techniques for establishing a neural workload index, as mentioned above, may also be employed in a closed-loop fashion. Section 7 demonstrates such a type of application in the context of adapting working environments to the human factor.

### 2.3. Comparisons of the categories of use cases

**Type I**. The use of BCIs as a tool for research is an easier case, in several ways. Experiments are conducted under laboratory conditions, complexity is often reduced to one variable of interest, standard EEG equipment with lengthy preparation times does not pose a serious problem, and it is not problematic if the system does not work for all participants. Still, specific requirements of a given use case can make the enterprise a challenge. In this category, the end users of the BCI are the researchers.

**Type II**. Using BCI technology in this way seems realistic in the medium term. The scenarios are often complex (e.g., driving a car), with mingled interactions between various perceptual and cognitive processes, calling for advanced decoding methods. On the other hand, there is no strict requirement for an easily deployable setup, the experimental conditions are somewhat controlled, and the experimental subjects can be selected according to the decodability of the given brain processes (provided that the selection criterion does not imply a bias with respect to the subject of investigation). Applications in this category mostly do not critically rely on the real-time aspect. In this case, the end users are the companies that develop the devices or interfaces, as well as the institutions that are responsible for the infrastructure.

**Type III**. BCI applications in this category are mostly rather far-reaching. The concepts are not yet ready for the dynamics and complexity of the real world, the measurement devices need to be easily deployable, yet also to provide robust signals, and the system should work preferably for anyone, as all human beings[PS3] are the end users in this category.

## 3. Detection of emergency braking intention during driving

This section summarizes two studies that investigate the possibility of predicting upcoming emergency braking from neural signals. The method makes use of exogenous ERPs related to perception and cognition in combination with an endogenous signal that indicates the preparation of a movement. We discuss potential benefits and caveats.

### 3.1. Context: neurotechnology in the context of driving a car

Neurotechnology can detect specific brain states before they reach consciousness and before they trigger behavioral actions (Section 7). Neuroergonomic approaches are therefore of interest for increasing our understanding of physiological aspects in time- and safety-critical applications, because a potentially dangerous situation may be detected before the user is aware of it and/or able to respond to it. In this section, we explore the feasibility and utility of this approach in driving a car. Kohlmorgen et al. (2007) and Dijksterhuis et al. (2013) study EEG correlates of mental workload during real-world and simulated driving, while (Kecklund and Åkerstedt, 1993; Papadelis et al., 2007; Schmidt et al., 2007, 2009; Gugler et al., 2010; Simon et al., 2011; Sonnleitner et al., 2012) study fatigue and attention during monotonous real-world driving. Reaction time in lane changing tasks has been investigated with EEG (Zhang et al., 2015a). Further studies demonstrate the detection of error and anticipatory potentials that could potentially be harnessed to increase driving safety (Khaliliardali et al., 2015; Zhang et al., 2015b). In emergency situations caused by obstacles on the road, drivers need to react quickly by braking. Such events lead to a cascade of mental responses from the perception and evaluation of the emergency-inducing stimulus to the activation of the lower limb muscles initiating the release of the gas pedal and the activation of the brake pedal. Due to the latencies inherent in motor responses, and due to the complexity of the required movement, the time spent between the stimulus and an effective deceleration of the vehicle can easily be on the order of 1 s, even if the decision to brake is made several hundred milliseconds earlier. This delay has led to attempts to retrieve the driver's braking intent earlier, which can be done by considering additional behavioral inputs such as gas pedal release, steering angle, foot position, and head movements (McCall and Trivedi, 2007; Trivedi and Cheng, 2007). Neuroergonomic approaches have the potential to prompt the user's decision even earlier—at the time of its very emergence—by tapping directly into the brain. This extra time might in principle either be used to prevent crashes through automatic braking, or to perform preparatory measures aimed at mitigating the impact of a crash through an automated response such as tightening the seat belts.

### 3.2. Two studies on predicting emergency braking

The first study to describe such a system was Haufe et al. (2011). In a driving simulator, *N* = 18 participants were instructed to follow a computer-controlled vehicle. The distance between vehicles was 20 m; the speed was 100 km/h. Occasionally (20–40 s inter-stimulus-interval, randomized), a rapid braking of the leading vehicle would induce an emergency braking situation. In order to avoid a crash, participants were required to perform immediate emergency braking in these situations. The onset of the lead vehicle's braking (and brake light flashing) is here referred to as the *stimulus*. The experiment comprised three blocks (45 min each) of driving with 10–15 min periods of rest in-between.

The authors measured myoelectric (EMG) activity using two bipolar electrodes located at the *M. tibialis anterior* and the knee of the right leg, as well as electroencephalography (EEG) from 64 scalp sites (nose reference). Behavioral and technical data—including brake and gas pedal deflections, the acceleration of the participant's vehicle, and the distance between vehicles—were also measured and synchronized with the physiological data. This yielded a multivariate, multi-modal time series from which the segments reflecting induced emergency situations and normal driving periods, respectively, were extracted.

Using univariate analysis, the authors discovered a characteristic event-related potential (ERP) signature preceding executed emergency braking in the EEG data. They then used multivariate machine learning to compare the predictive power (derived by distinguishing emergency braking from normal driving episodes) of a system utilizing all measurement modalities to systems either omitting EEG, EMG, or both. The study was later replicated with *N* = 20 subjects in a real car on a non-public test track (Haufe et al., 2014a).

**Figure 1:**
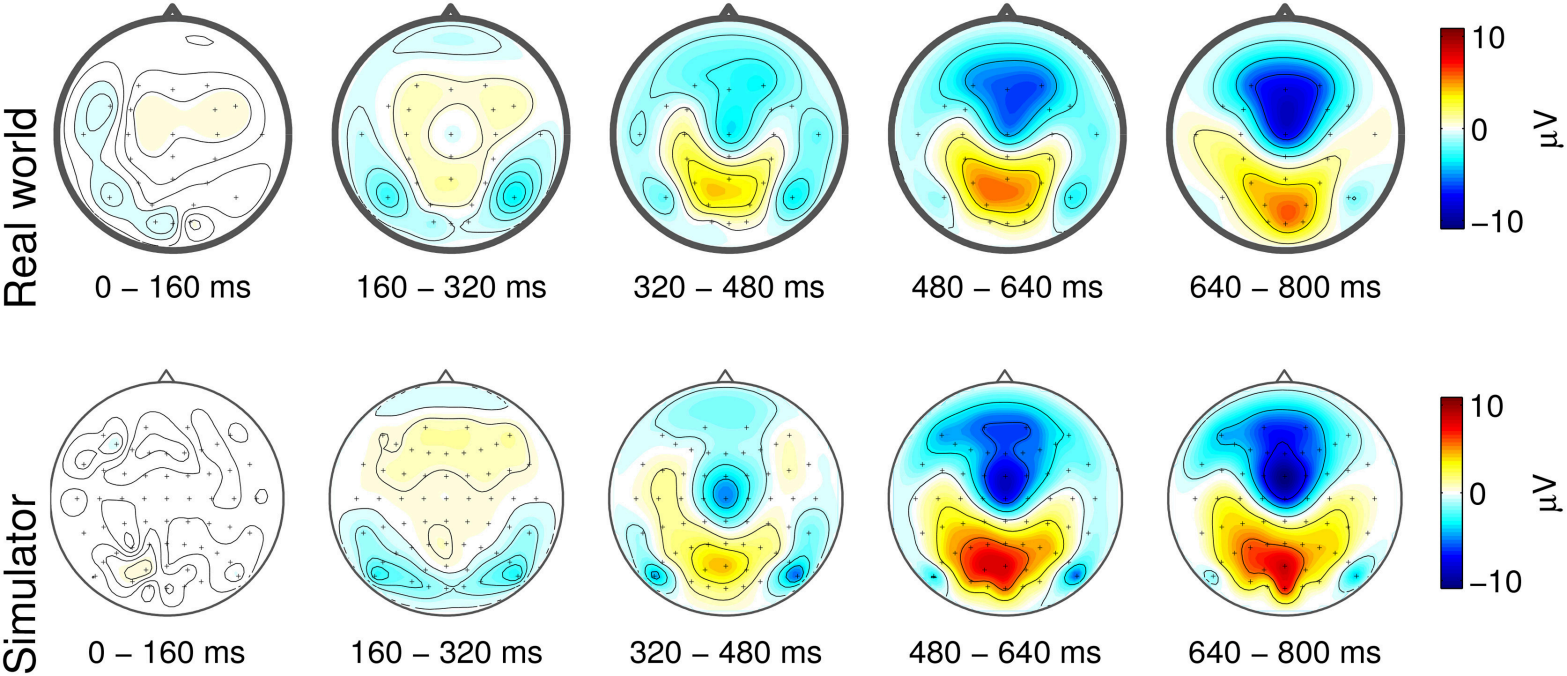
**Grand-average stimulus-aligned EEG responses to forced emergency braking during real-world (upper panel) and laboratory driving (lower panel)**. Potentials are visualized as topographical maps of grand-average ERPs in five temporal intervals. The stimulus onset (*t* = 0 ms) is the time of the brake lightbrake light flashing of the lead vehicle. Figure taken from Haufe et al. (2014a) with permission.

Figure 1 depicts the event-related potentials that are characteristic of forced emergency braking. In both studies, a spatio-temporal ERP complex composed of the three overlapping subcomponents was observed: an early symmetric negative deflection in occipito-temporal areas, a negativity at central scalp sites, and a positive deflection in centro-parietal areas. The early occipital negativity is a visual-evoked potential (VEP) that can be attributed to low-level processing of the flashing of the brake light of the leading vehicle, which initiates the emergency situation. Higher-level processing of the importance of this flashing is reflected in the later centro-parietal positivity (P300). The late central negativity amounts to the readiness potential (RP), which reflects the motor preparation and execution related to pressing the brake pedal.

**Figure 2:**
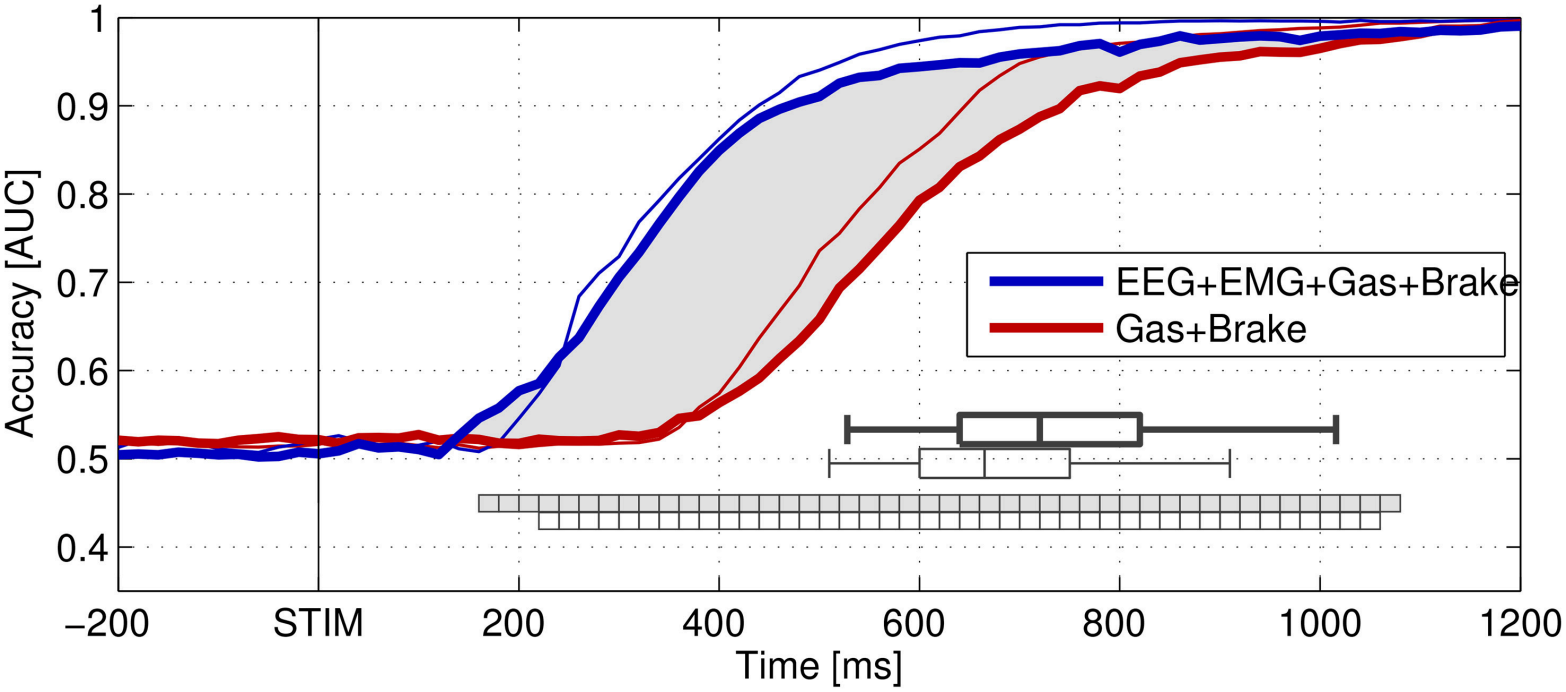
**Grand-average area under the curve (AUC) scores calculated from the outputs of linear classifiers that were optimized to distinguish normal driving intervals from stimulus-aligned target intervals representing different stages of emergency braking situations**. STIM denotes the onset of braking (brake light flashing) of the lead vehicle. Thick lines represent the results of the real-world driving study (Haufe et al., 2014a), while thin lines represent results obtained in the driving simulator study of Haufe et al. (2011). The distribution of pooled braking response times in both datasets is indicated by box plots showing the 5th, 25th, 50th (median), 75th, and 95th percentiles. Classification was based on (spatio-) temporal features observed prior to the decision points. Performance of combinations of modalities. Blue: EEG+EMG+Gas+Brake (electrophysiological and behavioral channels). Red: Gas+Brake (only behavioral channels). The intervals, in which the inclusion of electrophysiological channels significantly improved classification accuracy are marked as square boxes (no filling for simulated driving, light gray filling for real-world driving). Figure taken from Haufe et al. (2014a) with permission.

Figure 2 shows the detection accuracy of single-trial braking intention using a multivariate classifier as achieved on hold-out data and measured using the area under the curve (AUC) score. The classifier was evaluated at each stage of emergency braking between 0 and 1200 ms post-stimulus using data from the preceding 1500 ms. Without the EEG and EMG channels, the performance significantly dropped between approximately 200 and 1000 ms post-stimulus in both studies, indicating that EEG and EMG contain important information about the driver's intention that is not available in the combined remaining channels in the early stage of dealing with an emergency.

### 3.3. Conclusion and outlook

Summarizing, Haufe et al. (2011) showed that EEG and EMG recordings reach the predictive accuracy of behavioral channels at earlier stages of the emergency situation. Their laboratory results were reproduced during real-world driving (Haufe et al., 2014a), demonstrating that well-designed simulator studies can be a useful proxy for real world studies. The results have also been confirmed under more diversified traffic conditions (Kim et al., 2014; Khaliliardali et al., 2015).

The robustness of our findings motivates the use of neuroergonomic approaches to driving assistance. Such a system may detect a driver's intention to brake before any of their actions become observable, and may thereby reduce the time after which appropriate action can be carried out. Haufe et al. (2011) evaluated a simplistic implementation of an online emergency braking detector in their simulation environment, and estimated that the time that can be saved by the system is around 130 ms. At 100 km/h, this amounts to a reduction of the braking distance by 3.66 m. With respect to practical implementation, another aspect has also to be considered. While anticipatory brain signals allow for an early prediction of an action, they do not necessarily reflect the final decision as shown in the study discussed in Section 7. In the presence of motor predictive brain signals, the participant may still change their mind and cancel the movement or act differently. In the scenario of emergency situations, this could be caused by the insight that under the given condition the avoidance of an obstruction might be a better option than braking.

## 4. EEG-based classification of video quality perception

By the virtue of a specific experimental paradigm, the watchers' SSVEP amplitudes were modulated by degradations in the quality of videos. Classification methods derived neural indices that correlated with the mean opinion scores (MOS) given by the participants in the standard behavioral assessment, giving rise to a new approach to video quality assessment.

### 4.1. Context: brain-guided quality assessment

As we elaborated in our first review (Blankertz et al., 2010), there is a good outlook for using neurotechnology in usability studies because it allows for an effortless continuous acquisition of usability parameters without requiring any action on the part of the user. It may also include aspects that are difficult to quantify objectively with conventional methods and access variables unknown to the test subjects themselves. In a similar vein, neurotechnology may prove useful for the quality assessment of such multi-media content (Moldovan et al., 2013; Antons et al., 2014). Such an approach may capitalize on neural correlates of perceptual or cognitive processes. The assessment of audio quality based on EEG was pioneered in Porbadnigk et al. (2010). Their results showed that the methodology taken from BCI research has the potential to detect changes in the brain signals of listeners, who were presented with audio signals that had quality degradations below the threshold of perception, (cf. also Antons et al., 2012; Porbadnigk et al., 2013). This demonstrated an increased sensitivity compared to behavioral measures, which was also found in studies on the visual domain (Porbadnigk et al., 2011). In this section, we review studies investigating the neurotechnology-based assessment of video quality with the aim of improving video codecs.

### 4.2. Studies on neural measures of video quality

For the transmission of video signals at today's high bit rates, video codecs usually employ high levels of compression, which might introduce distortions visible to the human eye. It is therefore desirable to measure the perceived distortions through the assessment of the visual quality of compressed video. This assessment is usually done through so-called mean opinion scores (MOS) that are obtained through questionnaires, in which participants are asked to rate the quality of a visual stimulus on a rating scale (ITU, 2002, 2008). These behavioral tests have many limitations, including large inter-subject variance and the requirement of a high number of participants to achieve statistical significance. The judgment of the participants can also be biased by several factors not related to the quality of the stimulus itself. In recent years, therefore, there has been increasing interest in investigating novel paradigms for video quality assessment through the direct measurement of neural activity via EEG (Hayashi et al., 2000; Babiloni et al., 2006; Arndt et al., 2011; Lindemann et al., 2011; Mustafa et al., 2012; Arndt et al., 2014; Kroupi et al., 2014).

The authors of Scholler et al. (2012) used an experimental design that capitalized on the ERP component P3 to quantify the perception of the human observer when being confronted with a change in video quality. As stimuli, they showed 8 s video clips based on a synthetic image of a textured checkerboard, which was deformed over time by simulating a swaying water surface on the top. The quality change was introduced by lossy compression, while its magnitude was controlled by the quantization parameter of the video coder. Participants had to acknowledge the perception of the quality change via button press. They found that quality changes elicited a P3 component that was positively correlated with the magnitude of the change, which could be classified on a single-trial basis using LDA. They report a single-trial classification with AUC-values close to 1 for the highest level of distortion in most subjects, referring only to trials correctly identified by the participants at the behavioral level. They also report, for three participants, an average 65% accuracy in classifying the trials in which the quality change was present but not detected by the subjects, advancing the hypothesis of higher sensitivity of the EEG compared to the behavioral response.

All the previously mentioned studies are based on the detection of the P3 component, which is a cognitive ERP not directly linked to sensory processing. Complementarily, we investigated a paradigm based on Steady-State Visual Evoked Potentials (SSVEPs) that reflect perceptual processes. The basic suitability of the SSVEP-based design for video quality assessment has been demonstrated (Norcia et al., 2014). Here, we review a systematic follow-up study (Acqualagna et al., 2015).

**Figure 3:**
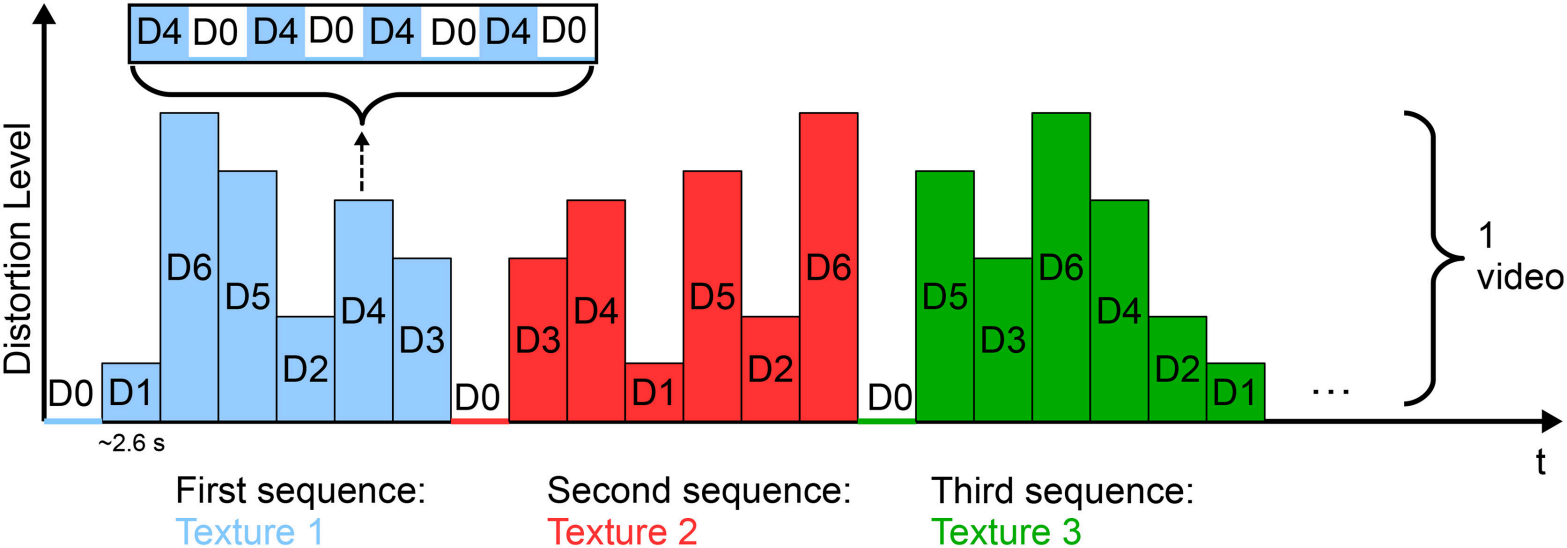
**SSVEP-based paradigm for video quality assessment**. Each video comprised the six textures presented in all the levels of distortion (D1, …, D6) in random order. Each texture was displayed distorted for 333 ms, followed by the undistorted form for 333 ms (D0) and the same succession was repeated four times for each level. Figure adapted from Acqualagna et al. (2015) with permission.

As stimuli, six gray-level natural images in six levels of degradation (corresponding to six compression rates) were used. The degradation levels were controlled by the quantization parameter, as in Scholler et al. (2012). The experiment comprised 51 videos in which all the textures in all the levels of degradation were displayed (Figure 3). The original and distorted textures were presented in alternating order with a stimulus onset asynchrony (SOA) of 333 ms (i.e., at a frequency of 3 Hz). The flickering effect of the sequence of quality changes caused the elicitation of SSVEPs in the occipital cortex, the amplitude of which increased with increased distortion levels.

**Figure 4:**
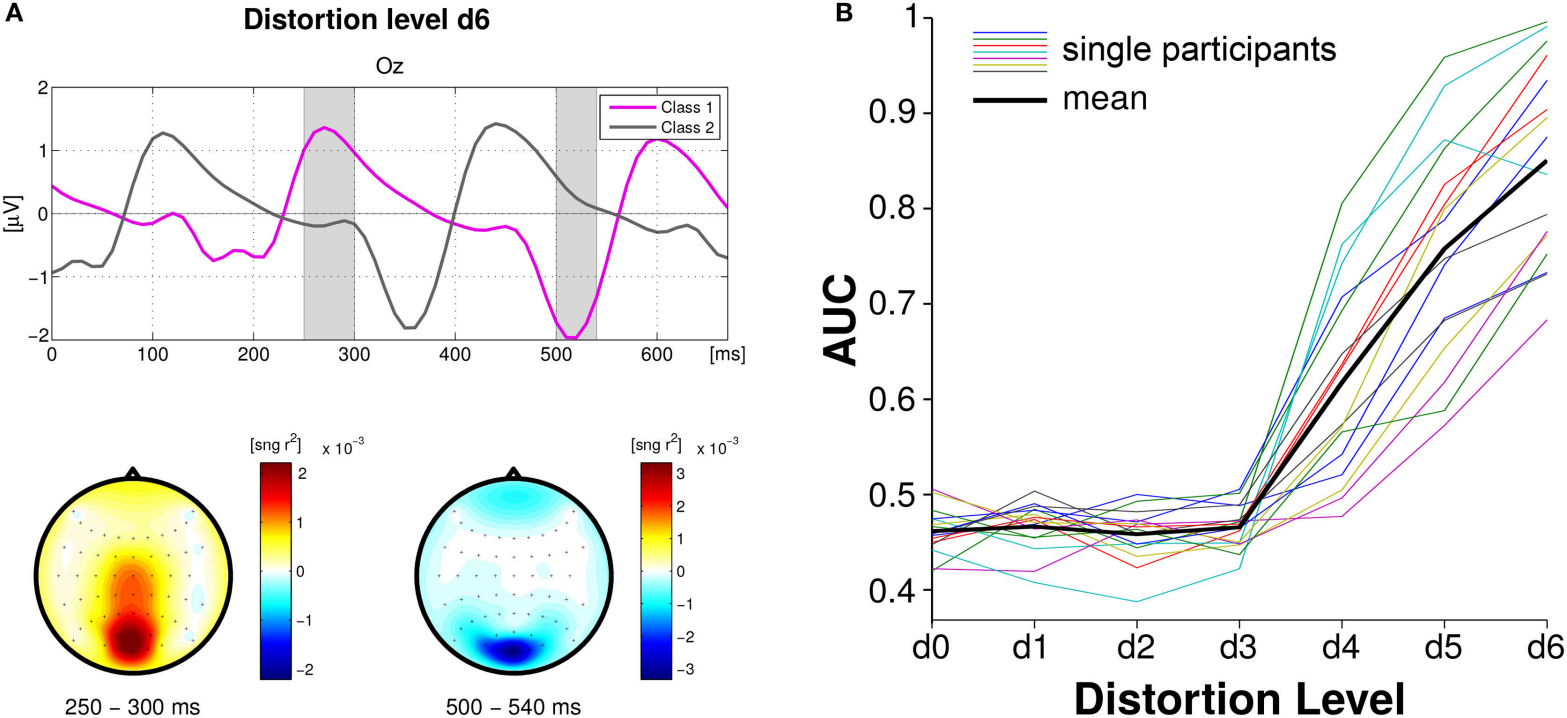
**Single-trial classification of VEPs based on spatio-temporal features. (A)** Grand average brain activity over all participants at channel Oz, at maximum distortion level D6. The magenta line represents “Class 1” and the gray line “Class 2.” Scalp plots underneath refer to the shaded areas in the time plot and display the magnitude of the *sign* − *r*^2^ for each channel. **(B)** Classification performances using shrinkage LDA for all participants (colored lines) and mean (black thick line). Figure adapted from Acqualagna et al. (2015) with permission.

In the first approach, single VEPs of the steady-state signal were classified using spatio-temporal features (Blankertz et al., 2011) applied to epochs without and with a time-lag of 160 ms (Figure 4, left). Single-trial classification achieved an average maximum AUC of 0.84 for the maximum distortion level. Figure 4 (right) shows that the first three distortion levels (D1–D3), which were chosen to be below the threshold of perception, did not modulate the SSVEPs and classification stayed at chance level. Further on, classification performance linearly increases with the distortion level. AUC scores significantly linearly correlated with MOS-values for all the participants (*p* < 0.01).

The same trend was obtained for the classification of spectral features at 3 and 6 Hz (i.e., the frequencies of the EEG spectrum that showed the highest modulation). CSP filters were calculated on the training data between the epochs referred to the maximum distortion level (D6) and those referred to the original textures (D0). CSP filters were then applied on the test data for all distortion levels. Features consisted of the log-variance of the CSP filtered data. Epochs locked to the distorted textures (D1–D6) were classified vs. epochs locked to D0 using LDA, achieving the maximum average AUC of 0.74 for D6.

### 4.3. Concluding remarks

Importantly, this study showed that an SSVEP-based paradigm allows a much quicker collection of trials than previous P3-based paradigms for quality assessment. The results of the neural assessment also correlated significantly with the MOS-values. The study thus demonstrated that an SSVEP-based video quality assessment can be considered a viable complement to behavioral-based assessments and a presumably faster alternative to methods based on the P3 component. For further details about the study and complementary analysis, please refer to Bosse et al. (2014, 2015) and Acqualagna et al. (2015). More generally, the results indicate the utility of the brain-based approach for usability testing and related quality assessment.

## 5. Advanced monitoring of workload

This section reports methodological advancements in the context of brain-based mental state monitoring (Blankertz et al., 2010) and neuroergonomics (Mehta and Parasuraman, 2013). We take the estimation of the so-called operator workload as an application scenario, although the methodology is much more widely applicable. We explored estimates based on spectral features of endogenous brain rhythms that differ with respect to the label information required for training, including entirely unsupervised approaches. The explored estimators also differed with respect to the level at which the spectral features are extracted, thereby comparing traditional single-channel based approaches to approaches that employ recent advances in spatial filtering methods (Schultze-Kraft et al., 2016b).

### 5.1. Context: neuroergonomics and physiology of operator workload

Many work places with high levels of automation require human operators to perform monotonous but attention-demanding tasks, such as driving and air traffic control, or as in industrial contexts. In such work environments, the demand for high levels of alertness can lead to an overload of the human operator, which in turn can have critical consequences for health, safety, and efficiency. An assessment of the operator's workload (Gevins et al., 1995; Gevins and Smith, 2003) can be utilized to prevent overload and can lead to adaptive systems that automatically self-regulate the level of human-machine interaction (Pope et al., 1995; Prinzel et al., 2003; Parasuraman and Wilson, 2008). Another area of application is in training procedures, in which displaying the current workload level to the trainer as well as to the trainee her/himself is expected to facilitate learning or to make the alignment of difficulty levels to the trainee's progress more efficient (Borghini et al., 2016). This possibility has been explored for pilots (Borghini et al., 2013), air traffic controllers (Di Flumeri et al., 2015), and shipmasters (Miklody et al., 2016). The technique could similarly be used to improve infrastructure, by testing, for example, which features of streets, harbors, and the like require maneuvers that are likely to induce high workload.

The human EEG has been shown to provide reliable estimators of workload, based on the fact that changes in workload are associated with characteristic modulations in the power of oscillatory activity in particular frequency bands of the EEG (Buzsáki and Draguhn, 2004). The most prominent frequency bands with power changes related to workload are theta (4–7 Hz) and alpha (8–12 Hz). Theta power has been shown to be positively correlated with workload, most notably in frontal regions (Gevins et al., 1998; Smith et al., 2001; Holm et al., 2009), whereas alpha power is typically found to be negatively correlated with workload, in parietal regions in particular (Gevins and Smith, 2003; Holm et al., 2009). This effect cannot be expected in general, however, as these results refer to the visual modality only (see the discussion Section 9).

### 5.2. Study comparing methodologies for estimating workload

**Figure 5:**
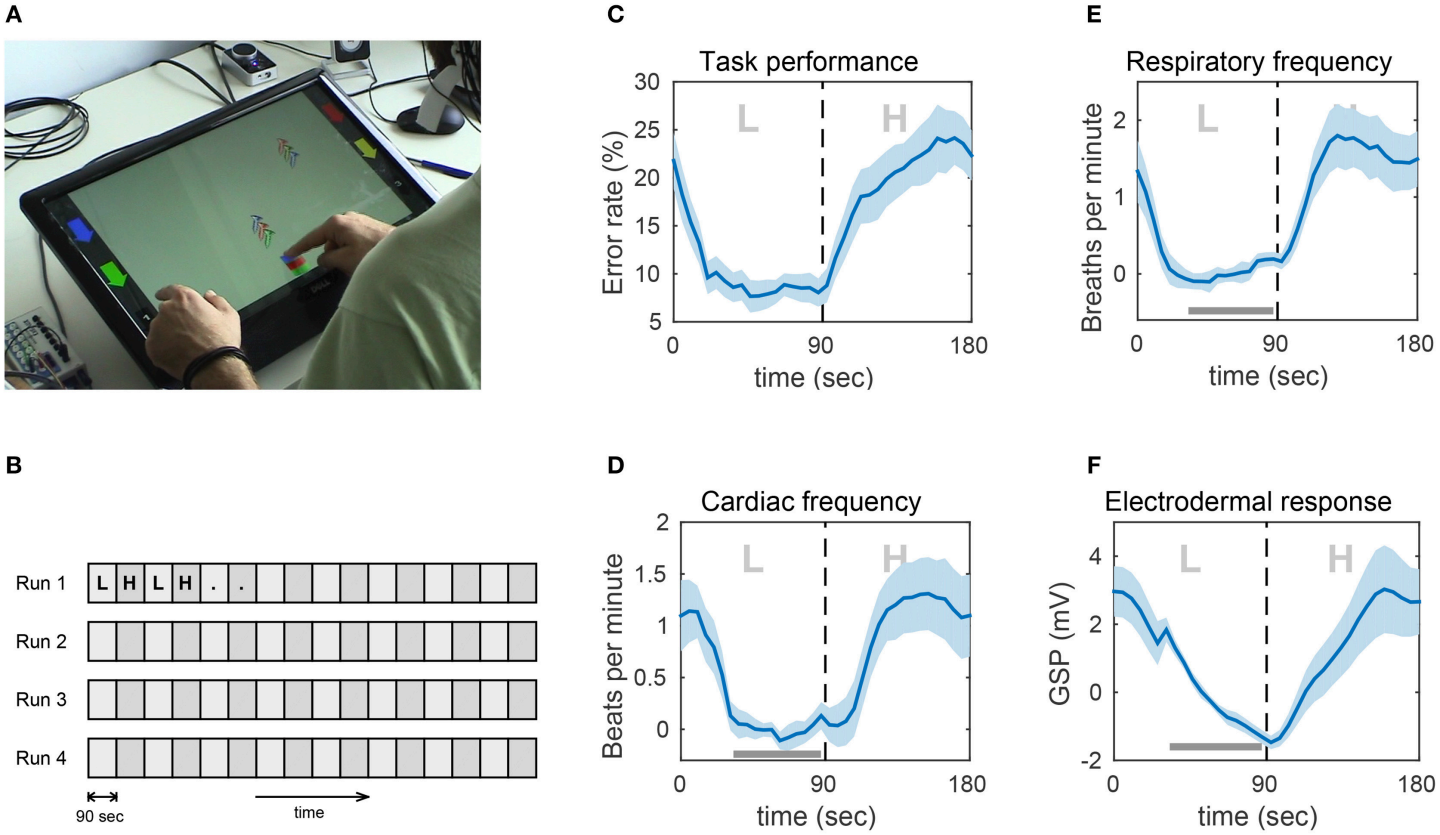
**Experimental task and impact of the experimental paradigm on task performance and peripheral physiological measures (PPM). (A)** Snapshot from one of the experiments showing a subject playing the game on the touch screen. **(B)** Block structure of the experiment. Participants performed four runs of 24 min, each consisting of 90-s blocks of alternating low (*L*) and high (*H*) workload conditions. **(C)** Error rate. **(D)** Respiratory frequency in breaths per minute. **(E)** Cardiac frequency in beats per minute. **(F)** Electrodermal response in Galvanic skin potential. Shown are the grand averages of the mean over all L–H block pairs. The light blue shadings indicate the standard error of the mean. Due to large inter-subject differences in the average of the PPMs, the grand average and standard error were computed after subtracting the mean in the indicated bar. Thus, the plotted values represent changes from this baseline. Figure taken from Schultze-Kraft et al. (2016b) with permission.

The experiment setup in Schultze-Kraft et al. (2016b) was designed to emulate an industrial working scenario in which an operator performs tasks requiring a continuous effort of visual and motor processing with alternating difficulty. Ten healthy male subjects, aged 26–40, participated in the experiments. Subjects were instructed to carry out a task on a 21-inch touch screen lying on a table in front of them (Figure 5A).

The task was designed as a computer game in which objects consisting of three vertically aligned screws (screw triplets) were falling from random positions at top of the screen and had to be caught in a bucket at the bottom of the screen. Subjects could move the bucket horizontally by sliding it with one of their index fingers. The coloring of the bucket could be adjusted by touching colored buttons that were positioned on either side of the screen. The catching task was further complicated by the constraint that the coloring of the bucket had to match the coloring of a screw triplet before catching it. Not catching a screw triplet was considered an error and so was catching a triplet with a color scheme that did not match the color scheme of the bucket. In the low workload condition (L), the interval between falling screw triples was constant, whereas in the high workload condition (H) the intervals were shorter and were randomly varied. The experiment was conducted in four runs, where each run lasted 24 min and consisted of alternating low and high workload blocks, with each block lasting 90 s. See Figure 5B for the experimental design.

In addition to 64-channel EEG, we also determined task performance (error rate) and measured the following peripheral physiological measures (PPM): respiratory frequency, cardiac frequency, and electrodermal response. See Figures 5C–F for the task-induced effects on error rate and PPMs. Switching from low to high workload induced consistent and significant increases in error rate and all PPMs.

In order to classify (or predict) workload levels, we compared six different predictive models. Three of them are based on spectral features at *channel-level*, whereas the other three used specific data-driven *spatial filters*. In the channel-level approaches, spectral features are computed for each recording channel separately. In the spatial-filter-level approaches, the data are first projected onto a set of optimized spatial filters. Spectral features are then computed based on the output of the spatial filters. Each of the channel-level and spatial-filter-level approaches fall into one out of three sub-categories, depending on the amount of information required by the approach. These sub-categories include (a) use of binary class labels (classification models, tag *cfy*), (b) use of a continuous error measure (regression models, tag *regr*), and (c) no use of a supervision signal at all (unsupervised models, tag *unsup*). In the classification models, spectral features were combined using regularized linear discriminant analysis (LDA) in combination with binary labels. In the regression models, spectral features were combined using regularized least-squares regression (LSR) in combination with the subject's error rate as a supervision signal. Finally, the output of the unsupervised models was simply the difference between theta and alpha features (Power diff). These three sub-categories represent a progression from (a) controlled laboratory conditions in which full label information is available to more realistic settings in which either (b) only a proxy-variable, such as the error rate is available, or (c) no external information about the variable of interest is available at all. This last scenario requires assumptions about the nature of the expected spectral changes in the EEG, which, in our case, is associated with workload (Gevins et al., 1998; Gevins and Smith, 2003; Holm et al., 2009).

Matching the level of additional information provided, we employed the following spatial filter methods. For the classification model, we used the Common Spatial Pattern (CSP) algorithm (Fukunaga, 1990; Koles, 1991; Blankertz et al., 2008) to train the spatial filters. For the regression model, we used the Source Power Co-modulation (SPoC) algorithm (Dähne et al., 2014a). For the unsupervised model, we used the canonical Source Power Co-modulation (cSPoC) algorithm (Dähne et al., 2014b). While CSP is a well-established method in the field of BCI, SPoC, and cSPoC represent recent advances in the development of spatial filtering methods. See Dähne et al. (2015) and Fazli et al. (2015) for further information on the background of these methods.

**Figure 6:**
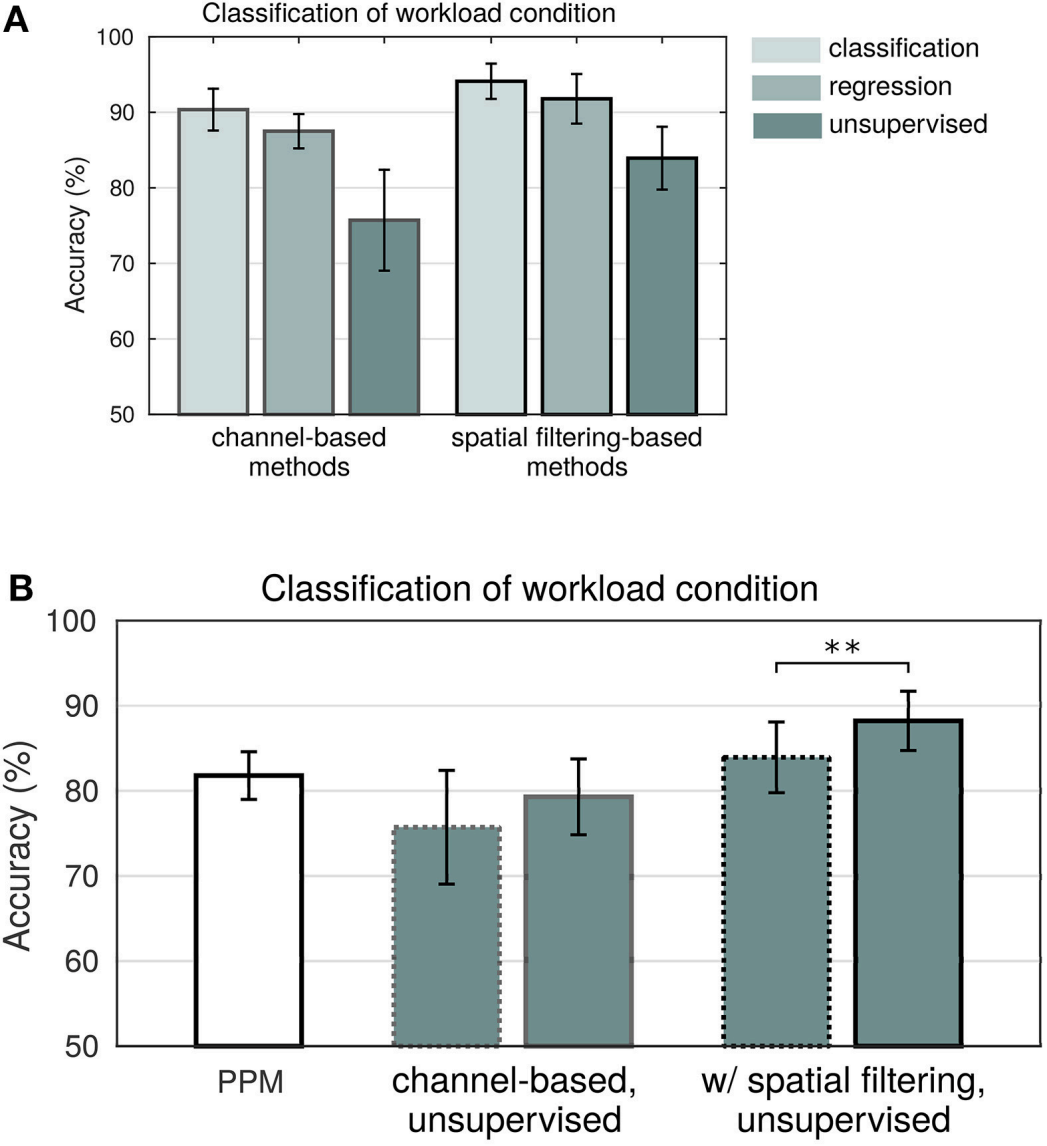
**Results**. **(A)** Mean classification accuracy and standard error of the six models across subjects. **(B)** Added value of peripheral physiological measures. Mean classification accuracy and standard error across subjects when using only PPM features (white) and comparison to the two unsupervised predictive models before (dotted) and after (solid) augmenting with PPM features. Figure taken from Schultze-Kraft et al. (2016b) with permission. ^**^Indicates a significance level of *p* < 0.01.

Figure 6A shows the mean classification accuracies for the six models, averaged across subjects. Both groups of models, with and without spatial filtering, show a decrease of performance with a decreasing amount of exploited label information. Between the groups, models using spatial filtering show a clear advantage that becomes greater the smaller the amount of label information that is available: 3.8% (CSP), 4.2% (SPoC), and 8.2% (cSPoC), compared to the respective channel-based method.

Given the modulation of PPMs by the workload condition (Figures 5C,D) and given that PPM features can be extracted from the data as an unsupervised signal, we assessed whether PPM features constitute an added value to the features extracted in the unsupervised models. We first of all found that the mean classification accuracy using only PPM features was 81.8% (Figure 6B, white bar). We then repeated the analysis with unsupervised models. This time, however, we augmented the EEG features with PPM features. This resulted in enhanced classification accuracies: a 3.6% increase for the channel-based (n.s.) and a significant increase of 4.3% for the spatial filtering-based method. Note that the unsupervised but PPM- augmented model outperformed the supervised channel-based model and even nearly equaled the performance of the supervised model, which used spatial filters. These results support the assumption that peripheral physiology can indeed provide an added value to the unsupervised model for the classification of workload.

**Figure 7:**
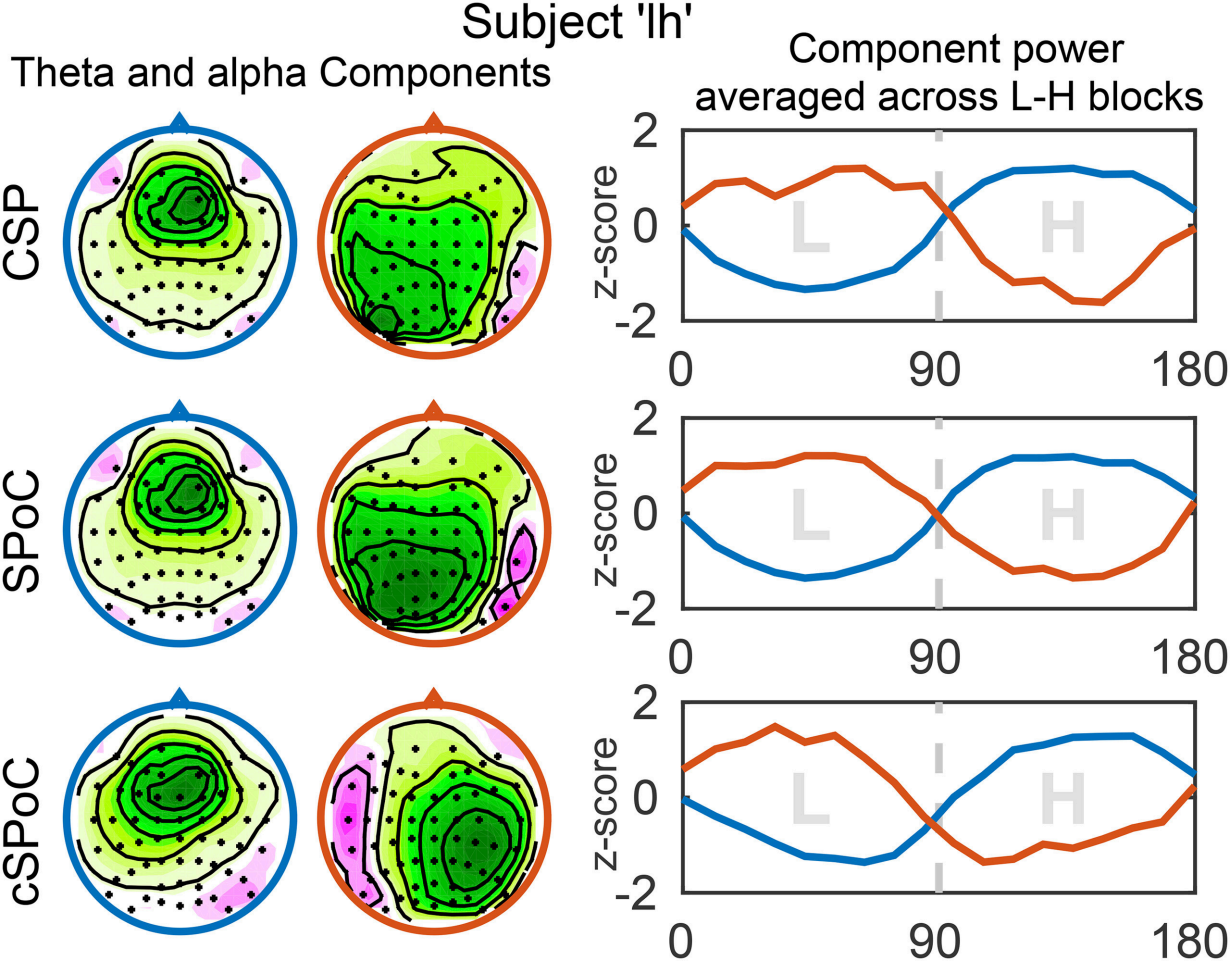
**Spatial activation patterns and power envelopes of components extracted by the three EEG spatial filtering methods**. The shown activation patterns (scalp maps) and power envelopes correspond to the components with the highest value of the optimization criterion of the respective method. The left and middle column show the activation patterns of components obtained from the theta (blue) and alpha (red) bandpassed data, respectively. The color coding and sign of the activation patterns were adjusted to be consistent across methods, but are arbitrary otherwise. The power envelopes (right column) are color-coded accordingly (theta: blue; alpha: red), the x-axis shows time in seconds. Due to standardizing to *z*-scores, the amplitudes of the curves do not relate to discriminative power. Figure taken from Schultze-Kraft et al. (2016b) with permission.

Next to an increased signal-to-noise ratio, a further benefit of using spatial filtering methods is that they allow for the inspection and interpretation of spatial activation patterns, which are associated with the extracted signals (Haufe et al., 2014b). It is thus possible to verify that the signals extracted by the CSP, SPoC, and cSPoC filters were of cortical origin, as opposed to possibly stemming from ocular or other artifactual sources. For this purpose, we examined the spatial activation patterns that correspond to the components found using the three methods, as well as the corresponding power envelopes of the components' time series. Figure 7 shows the activation patterns and corresponding power envelopes for one example subject. The activation patterns indicate that frontal theta activity and parietal/occipital alpha activity are the sources of workload modulated signals.

### 5.3. Conclusion and outlook

In summary, we find that using established and, in particular, recently developed spatial filtering methods, it has become possible to estimate an operator's workload based on brain signals and peripheral measures. Additionally, we would like to point out that we observed similarly promising results in a workload-related study (Naumann et al., 2016) in which subjects played the classical Tetris video game. In contrast to most workload-related studies, the task in Naumann et al. (2016) was not to classify workload into one of two categories (typically high vs. low workload). Instead, we aimed to predict the player's current game level, purely on the basis of spectral features from the brain. We employed the model outlined above (SPoC combined with regression) and we were able to predict the current gaming level with high precision (Naumann et al., 2016), results that are comparable with the findings presented in Schultze-Kraft et al. (2016b).

## 6. Brain-computer interfaces for human-computer interaction

While intentional control of computer applications is the target of classic BCI research for patients, this approach does not seem promising for general users. Instead, it seems worthwhile to employ BCIs to infer implicit information during software usage and to use that information to augment the explicit interaction. In other words, to make the computer better at understanding the human user on the basis of soft skills. In view of this far-reaching goal, we have investigated several studies that pave the way.

### 6.1. Context: BCIs for general human-computer interaction

The interaction with a complex interface might be facilitated if the system is able to exploit implicit information about the cognitive state of its user inferred from physiological signals. Measures from eye movement patterns, pupil size, electrodermal activity (EDA), facial electromyography (fEMG), and other peripheral physiological signals can provide insights into the user's mind with respect to relevance, attention, or intent (Oliveira et al., 2009; Hardoon and Pasupa, 2010; Cole et al., 2011a,b; Gwizdka and Cole, 2011; Haji Mirza et al., 2011; Hajimirza et al., 2012; Barral et al., 2015). However, electrophysiology may provide a more direct access to the cognition of the user in comparison to eye tracking or peripheral physiology (Zander and Kothe, 2011; Eugster et al., 2014; Ušćumlić and Blankertz, 2016; Wenzel et al., 2016).

Transferring the decoding results from the classic (fixed-gaze) BCI systems toward general human-computer interaction (HCI) applications, in which complex displays are explored in an unconstrained free-viewing manner, holds a number of challenges that we discuss in this section. These scenarios, which allow natural behavior, essentially require co-registration of eye-movements and EEG, since free viewing implies self-paced scene exploration. The emerging research on eye fixation-related potentials has shown that fixations on the object of search evoke EEG responses similar to the one evoked in the classical visual oddball paradigm with fixed gaze (Rämä and Baccino, 2010; Kamienkowski et al., 2012; Brouwer et al., 2013; Kaunitz et al., 2014), which motivated an expansion of potential BCI applications. The joint EEG and eye-tracking studies, such as those addressing active visual search of the target face in images of crowds (Kaunitz et al., 2014), or during navigation of 3D naturalistic environments (Jangraw et al., 2014), provide evidence for the feasibility of EEG-based intention decoding in realistic active visual search tasks. Both studies, however, impose certain constraints, either in terms of subjects' behavior (i.e., promoting longer fixations Kaunitz et al., 2014) or in terms of stimuli that prevent the overlap of target responses (Jangraw et al., 2014). Neither of these studies, moreover, address the system's performance with respect to the content of scenes, i.e., its semantic and spatial distribution, clutter, and temporal dynamic.

We conducted various experiments to approach these issues, taking as a guiding example the assessment of the relevance of the items on screen with regard to the user's task (e.g., information seeking). The goal is to obtain implicit information about the user's intention from the brain signals (as co-registered eye-tracker data) to supplement the explicit interaction with computer software via mouse and keyboard. Implicit relevance measures can be captured unobtrusively in the background and consume less time and effort in comparison to a laborious manual evaluation of the relevance of each item. In this transfer of BCI technology to realistic settings of human-computer interaction, we face a number of challenges that we address in the following section.

### 6.2. Variable neural latency in dynamic scenes

**Figure 8:**
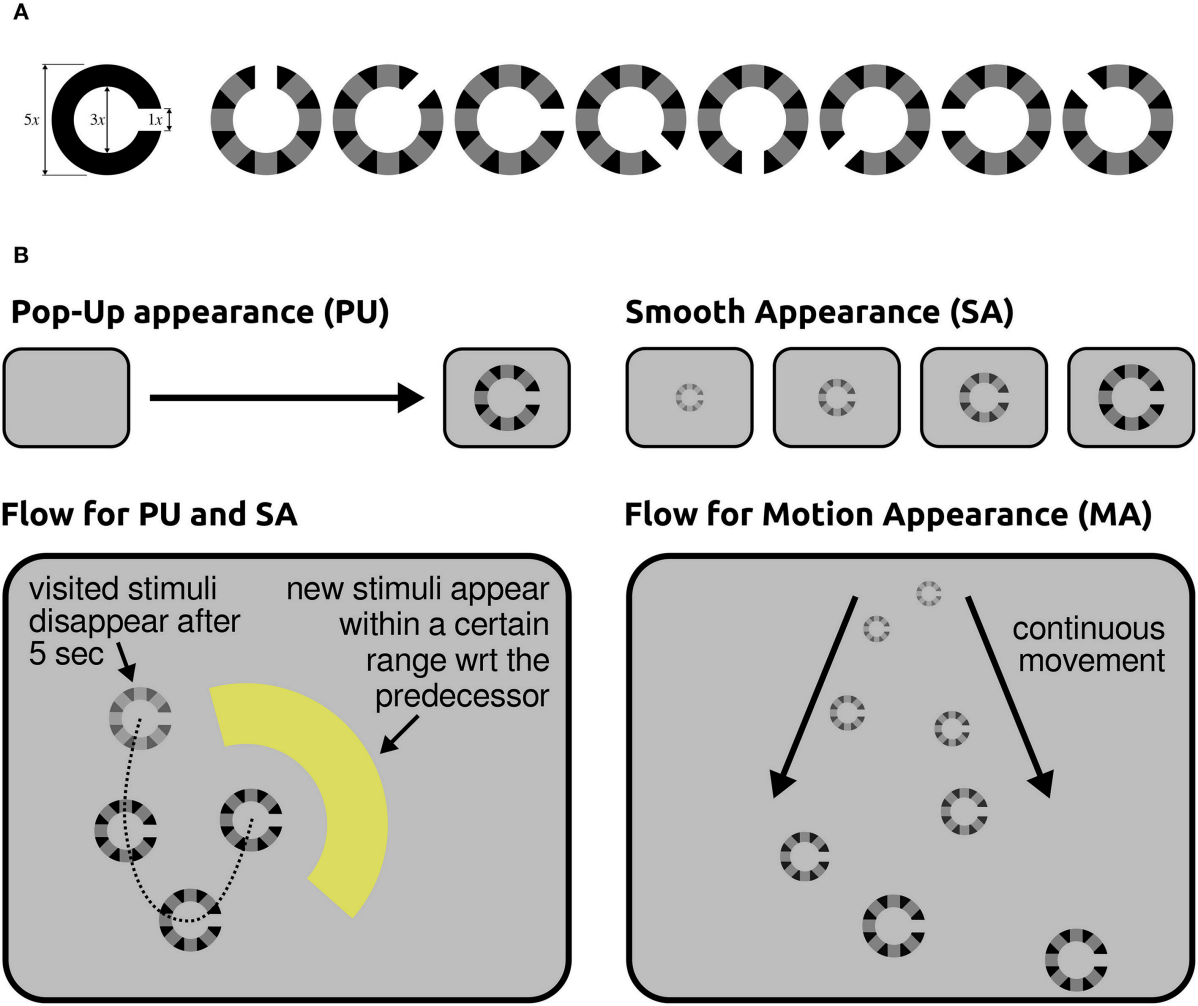
**(A) Left:** A standard Landolt broken ring. **Right:** Eight modified Landolt rings that we used in our study. **(B)** Illustration of the stimuli presentation flow for different conditions. **Top-left:** In the PU condition, stimuli appear in one step. **Top-right:** Three intermediate steps of stimulus evolution in time are presented for the SA condition, followed by a completely revealed stimulus. **Bottom-right** The dashed line indicates the order of the appearance of stimuli in the PU and SA conditions. **Bottom-left** Several intermediate steps in the evolution of two successive stimuli are illustrated for the MA condition. The arrows indicate the direction of their continuous motion. This illustration is simplified, since multiple objects were present on the screen during the motion condition (MA). Stimuli are enlarged in comparison to the real screen dimensions. Figure taken from Ušćumlić and Blankertz (2016) with permission.

**Figure 9:**
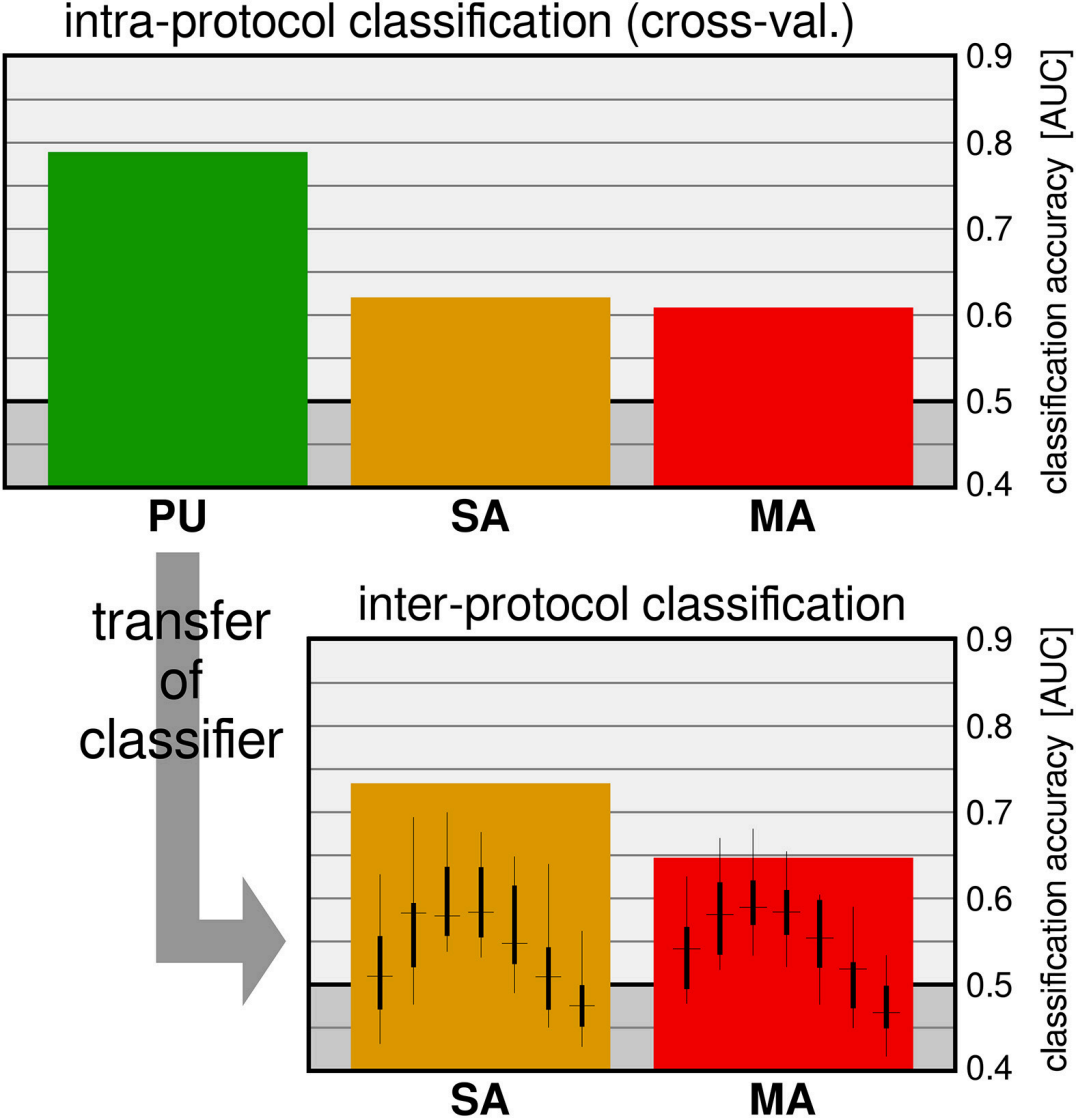
**Upper plot:** Intra-protocol classification with an HDCA was cross-validated on the three conditions. **Lower plot:** For the inter-protocol classifier transfer, the HDCA was trained on the training data (PU condition) on a fixed time interval. The intermediate results of the testing datasets (conditions SA and MA), obtained for different positions of the sliding window (small boxplots), are combined to give the results indicated by the broad bars. Figure adapted from Ušćumlić and Blankertz (2016).

On the one hand, applications for information seeking and retrieval are nowadays characterized by rich and dynamic visual interfaces. Such interfaces inevitably engage different attentional and perceptual brain processes (e.g., covert attention and peripheral vision) to sample the relevant content of scenes through consecutive eye-fixations. On the other hand, information is typically provided as semantic concepts that are richer than a simple symbol or a plain word; they may be contained in data of different modalities (e.g., text, image, and video). While a semantic concept may be ambiguous or vague, its recognition may require integration of evidence over time. Typical examples of the latter case are action and behavior recognition, or the recognition of new content on a screen when transition visual effects are applied, as it is often the case in visual interfaces. Altogether, in real world applications the cognitive processing may vary in both duration and onset time with reference to fixations. Our recently published study (Ušćumlić and Blankertz, 2016), which was motivated by the non-stationarity of our natural visual environment, addressed the EEG correlates of visual recognition while participants overtly performed visual search in non-stationary scenes. Our research particularly concerned whether scene dynamics might intensify the temporal uncertainty of ERPs with reference to fixations, introducing an extra challenge for state-of-the-art EEG decoding methods. We designed three free-view visual search tasks mimicking the type of visual effects that may appear in real world human-computer visual interfaces. Alongside popping-up stimuli, two composite appearance styles based on fading-in, enlarging, and motion effects were considered (Figure 8). In the Pop-Up (PU) and Smooth Appearance (SA) conditions, stimuli appear at random but fixed positions on the screen. In the Motion Appearance (MA) condition, the constant stimuli motion causes the entire scene to continuously change (for details, see Ušćumlić and Blankertz, 2016). First, we investigated the performance of the state-of-the-art EEG decoding method *hierarchical discriminant components analysis (HDCA)* (Gerson et al., 2006) across different conditions. The results confirmed our concerns, indicating a drop in decoding performance when facing less certain timing of visual events (i.e., the appearance of new content in a scene) due to the transitional changes (cf. Figure 9 upper panel). We showed, however, that the knowledge obtained from the paradigm characterized by less temporal uncertainty (i.e., popping-up stimuli) can be exploited to boost the EEG decoding performance in more challenging conditions. This is done by estimating posterior probabilities across different time lags with reference to fixation onset, using the classifier trained on popping-up condition, and by making the final decision based on a maximum of the estimated posteriors. The improved decoding performance, estimated in a 10-fold validation setting, is presented in Figure 9 (lower panel), for fading-in and motion conditions, respectively.

### 6.3. Variable neural latency due to variable saliency

**Figure 10:**
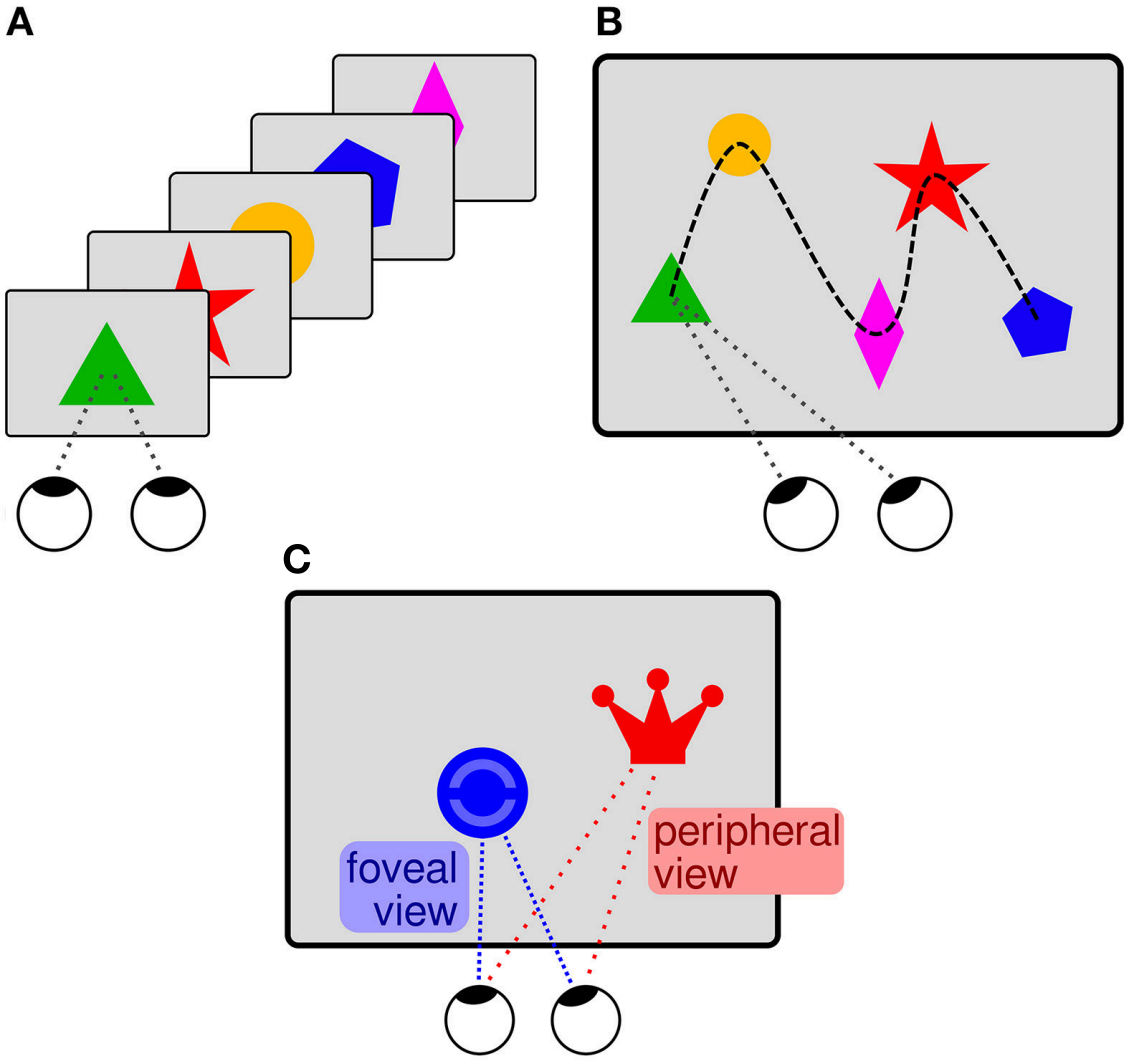
**(A)** Sequential centered “BCI presentation.” A simplified “mental typewriter” serves as example for an ERP-based BCI (cf. Treder et al., 2011). Different items (square, triangle, disc, pentagon) are flashed one-by-one, on the same spot on the screen. Each item stands for a (group of) letter(s). The subject selects a letter and silently counts the flashes of the corresponding item in order to direct their attention toward it. The selected (group of) letter(s) can be decoded from the EEG data using the flashes as time points of reference. **(B)** Item scanning in free-viewing. In an HCI scenario, words or pictograms (symbolized in the illustration) are displayed in parallel on the screen and are not flashed one-by-one. Items of particular interest for the user shall be decoded from the EEG data. The saccades (white arrow) to the items, as measured with an eye tracker, can serve as time points of reference for the EEG analysis. **(C)** The stimulus saliency may vary in HCI settings. An item of little saliency (here represented by a blue disc with indistinct interior) can only be recognized after a saccade when the item is in foveal vision. In contrast, a salient item (red crown) can be recognized already in peripheral vision, before a saccade toward it. A variable timing of recognition can therefore be expected with respect to the saccades, which are used as time points of reference for the EEG analysis.

In typical BCI experiments, different stimuli are flashed one-by-one (cf. Figure 10A) and the EEG is segmented in stimulus-aligned epochs that are used to predict the selected stimulus of interest (e.g., Treder et al., 2011). In regular software applications, several items are usually presented in parallel, rather than one-by-one. In this case, the saccades to the items, as measured with an eye tracker, can serve as time points of reference for the EEG segmentation (cf. Figure 10B). Using this approach, it is possible to estimate which items displayed on the screen are task-relevant and which are not (e.g., Brouwer et al., 2013; Kaunitz et al., 2014; Ušćumlić and Blankertz, 2016; Wenzel et al., 2016).

Pictograms and words shown in real software applications are usually diverse and feature different colors, shapes, and sizes. Saliency, which enables the recognition of relevant items, varies accordingly, such that recognition can happen either before the saccade (when the item is still in peripheral vision) or after the saccade to the item (when the item is in foveal vision; cf. Figure 10C). Accordingly, neural activity related to recognition can exhibit a temporal variability with respect to the saccades, which are used as time point of reference for the EEG segmentation. BCI prediction algorithms are not required to deal with this temporal variability because the stimulus onset serves as reference and the eyes are not moved in typical BCI experiments. An experiment with unrestricted eye gaze was performed in order to systematically investigate whether the algorithms can cope with this issue, which can be expected in realistic HCI settings (Wenzel et al., 2016). The participants were asked to find and count certain items that were presented in parallel on the screen and that were sometimes more and sometimes less salient. The continuous EEG data were segmented in epochs aligned to the (ends of the) saccades toward the items. Salient task-relevant items evoked an earlier neural response in comparison to less salient task-relevant items, presumably because recognition was possible already in peripheral vision. Nevertheless, even when the item saliency was mixed, a typical BCI prediction algorithm was suited to deal with the resulting temporal variability and was able to detect the task-relevant items in this search task.

### 6.4. Interference of eye movements with the EEG

EEG epochs used for the predictions in BCI experiments are usually set at several hundred milliseconds long in order to capture the P300 wave. But fixations often last only few hundred milliseconds and the subsequent saccade can occur during the same EEG epoch. This is problematic because eye movements can interfere with the EEG data. Yet, even when the eye movements were unrestricted, it was possible to capture neural signals related to recognition when the discriminative information was not (primarily) a result of eye movements (Wenzel et al., 2016). Interestingly, information from the two modalities of EEG and eye tracking were found to be complementary. An investigation of target detection with moving objects that require smooth-pursuit eye-movements showed no decrease of decoding performance if the timing of the event was known (Ušćumlić et al., 2015).

### 6.5. Insufficient accuracy in single-trials

EEG and eye tracking data can be informative about item relevance, but they are not sufficient for a reliable relevance estimate after a single fixation of an item. Brain-Computer Interfaces have to deal with a similar uncertainty and address this problem usually by combining predictions from several EEG epochs for class selection. The same strategy could be followed for the transfer to human-computer interaction. Practical applications should be designed such that evidence about user relevance is accumulated over time. This approach is favored naturally, because humans frequently move their eyes, which may result in a large number of saccade-aligned EEG epochs. For instance, when subjects scanned a mosaic of images that belonged to two classes, it was possible to reliably estimate, based on EEG and eye tracking data, which of the two classes of images was more relevant for the user, even if the predictions for the single images were not accurate. This strategy allowed the implicit resolution of ambiguities in an image web search (Golenia et al., 2015).

### 6.6. Conclusion and outlook

Taken together, these results indicate the basic feasibility of exploiting implicit information through the use of BCI techniques for human-computer interaction. This holds also if the targets are not previously known stimuli, but rather semantically described categories consisting of a large variety of previously unseen stimuli (Acqualagna and Blankertz, 2015). Apart from relevance, estimating the “depth of cognitive processing” would be useful in HCI when, for example, interfaces can adapt according to whether displayed information was adequately processed by the user or not. Recent work indicates that this variable can also be estimated sufficiently from the EEG (Nicolae et al., 2015a,b).

For a realistic perspective of using BCI technology in general human-computer interaction, the extraction of implicit information needs to be further improved, as in the context of, for example, more complex visual stimuli (Wenzel et al., 2015). Moreover, the co-registered acquisition of EEG and eye tracking data needs to be simplified to a deployable setup. We briefly discuss this aspect in a paragraph in the concluding Section 9.

## 7. BCI as a research tool in cognitive neuroscience

Employing a BCI to obtain early predictions of motor intentions in a gaming scenario required participants to cancel self-initiated button presses upon seeing a stop signal. By virtue of this paradigm, conclusions about the deterministic coupling between preparatory brain signals and the corresponding motor actions could be drawn that have a number of important implications ranging from the debate over free will to ethical considerations about applications that potentially speed up human behavior.

### 7.1. Context: preparatory signals and research questions in cognitive neuroscience

The readiness potential (RP) is a slow, negative cortical potential that is observed over motor areas in the EEG and can start more than one second before voluntary, self-initiated movements (Kornhuber and Deecke, 1965). It gained particular fame in the work of Libet et al. (1983), who found that the conscious decision to move occurs several hundred milliseconds after the onset of the RP, thereby initiating a still-ongoing heated debate about free will (Libet, 1985). One particular question that has remained unanswered is whether a person can still exert a veto by inhibiting a voluntary movement after onset of the RP (Haynes, 2011). One possibility is that once that RP begins to build up the planned movement must occur and cannot be canceled (De Jong et al., 1990). Another possibility is that people can still exert a veto by canceling or altering the movement after the onset of the RP. If the latter is the case, a follow-up question is whether there exists a point of no return along the time course of the RP, after which people cannot stop the planned movement. In order to test this, we devised an experiment that required subjects to cancel a self-paced movement once an RP had been detected by a BCI in real time.

### 7.2. Study on the coupling of preparatory signals and corresponding actions

The experimental task was designed as a “duel” between the subject and the computer. Subjects (*N* = 10) were confronted with a floor-mounted button and a light presented on a computer screen. If the subject pressed the button while the light on the screen was green, they would win a point. If they pressed the button after the computer had turned the light red (stop signal), they would lose a point. The experiment had three consecutive stages. In stage I, stop signals were elicited at random onset times. The EEG data from stage I were then used to train a classifier to predict upcoming movements. In stages II and III, movement predictions were made in real time by the BCI with the aim of turning on the stop signal in time to interrupt the subject's movement. EEG signals were continuously classified by the Berlin Brain-Computer Interface toolbox (https://github.com/bbci/bbci_public) in order to control the stop signal. Additionally, EMG was recorded from the calf muscle of the moving foot in order to determine the time of movement onset. For details on experimental procedures, please refer to Schultze-Kraft et al. (2016a).

**Figure 11:**
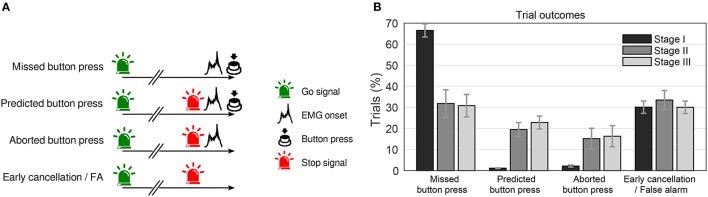
**(A)** Possible trial outcomes during the experiment. See text for details. **(B)** Percentage of trial outcomes across stages for the four trial categories (as in panel **(A)**). All trial categories in one stage (bars of same color) add up to 100%. Shown is the average across subjects (error bars = SEM). Figure taken from Schultze-Kraft et al. (2016a) with permission.

Each trial could end in one of four possible ways (Figure 11A): In “missed button press” trials subjects, won a point when they pressed the button while the light was green, whereas in “predicted button press” trials, they lost a point when they pressed the button after the stop signal had been turned on. Another possibility is that the BCI indicated an RP, elicited a stop signal, and the subject started to move (as indicated by EMG activity), but canceled the movement early enough (“aborted button press” trials). In the last case, the stop signal was elicited, but the participant showed no overt sign of movement. This trial type is ambiguous because it could either result from a prepared movement being terminated at an early stage (“early cancelation”) or it could reflect false positive detections by the classifier (“false alarm”).

We first of all examined the efficiency of the BCI predictor to detect RPs and elicit stop signals in real time. Figure 11B shows that while during stage I (random predictions), roughly 2 out of 3 trials were “missed button press” trials, during stages II and III only 1 out of 3 button presses were missed. Furthermore, predicted or aborted button press trials occurred very rarely during stage I, while during stages II and III they occurred in roughly 20 and 15% of trials, respectively. “Early cancelation/false alarm” trials occurred at comparable rates in all three stages.

**Figure 12:**
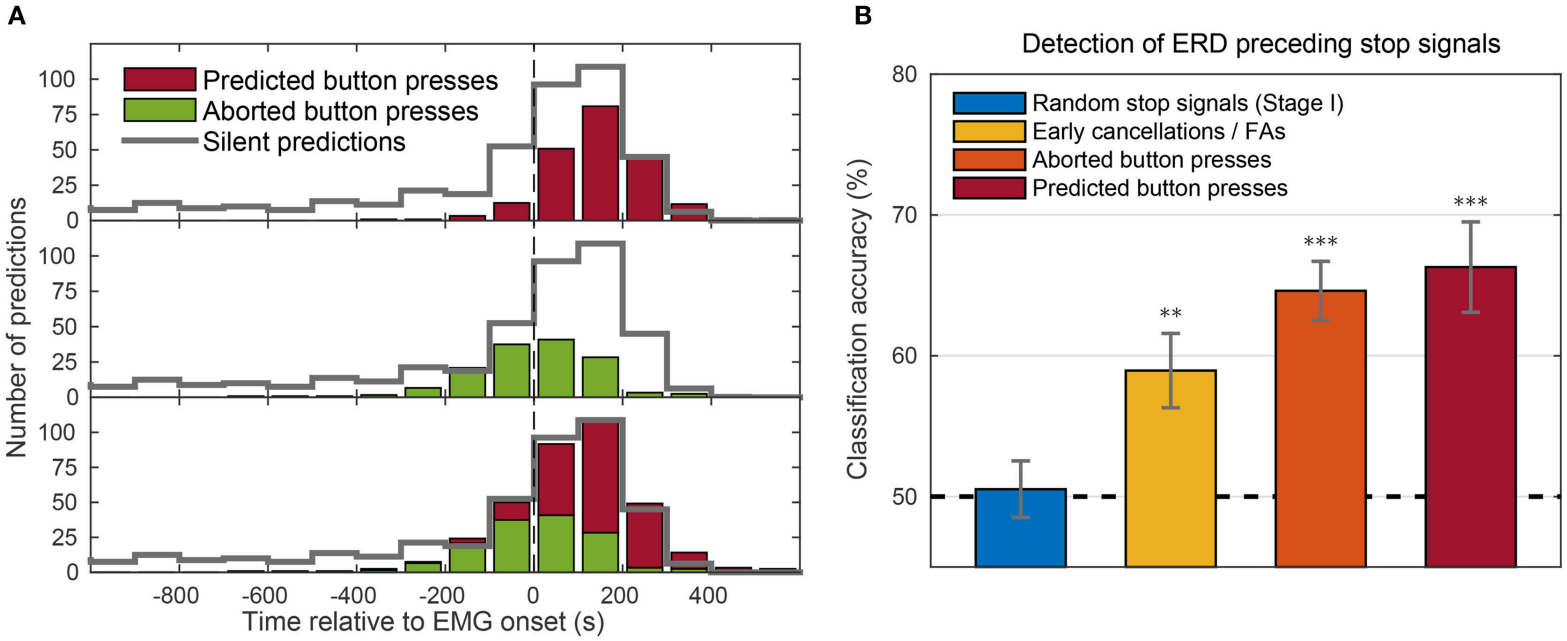
**(A)** Distribution of BCI predictions time-locked to EMG onset (vertical line). The three panels show the distribution of stop signal timings in predicted button press trials (top, red) and in aborted button press trials (middle, green). The bottom panel (red and green) shows their joint distribution. The gray distribution superimposed as outline in all three panels shows the stop signal distribution in silent trials, adjusted to account for the imbalanced probability of a trial being silent (40%) or not (60%). All bins comprised intervals of 100 ms and counts were pooled across stages II and III of all subjects. **(B)** Accuracies of a classifier trained to detect an impending movement based on event-related desynchronization (ERD) occurring before stop signals. Bars show the mean accuracies of subjects (error bars = SEM) for four different trial types. Figure taken from Schultze-Kraft et al. (2016a) with permission. Significance above chance level is indicated by ^**^p < 0.01 and ^***^p < 0.0001.

Next, we assessed how the timing of stop signals was related to movement onsets (as assessed by EMG). The distribution of stop signals in “predicted button press” trials (Figure 12A, red, top panel) shows that the vast majority of stop signals occurred after EMG onset. Since the movement was completed by pressing the button, the stop signal presumably came too late for a veto. Stop signals in “aborted button press” trials (Figure 12A, green, middle panel) occurred earlier (starting around 200 ms before EMG). Thus, when stop signals were presented at late stages of movement, preparation subjects could not stop themselves from beginning to move, even though they could abort the movement, once started. There was a gradual transition between stop signal times in which movements could be aborted and those in which they could not be aborted (Figure 12A, bottom panel).

It is interesting that there were rarely any cases in which subjects moved despite seeing stop signals earlier than 200 ms before EMG, even though RP onset occurred more than 1000 ms before EMG onset. We therefore examined the timing of predictions in “silent trials,” which occurred in 40% of trials during stages II and III. Here, when the BCI predicted a movement, the time was silently recorded, but the stop signal was not turned on and the trial continued until the button was pressed. As a detailed investigation of those silent trials shows (cf., Schultze-Kraft et al., 2016a), although a majority of predictions also in silent trials occurred around movement onset, many silent predictions occurred more than 200 ms before movement onset. The fact that these early predictions were absent for predicted button press trials or aborted button press trials suggests that the BCI was indeed able to predict movements at such early stages and that subjects were caught early enough to cancel their decision without any overt sign of movement.

In order to further investigate this assumption and assert whether predictions in the ambiguous trial type were early cancelations or false alarms, we looked for the occurrence of event-related desynchronization (ERD) in these ambiguous trials at the time of prediction. ERD occurs before and during movements in particular frequency bands in the EEG and has been shown to have a different generator in the brain than the RP, therefore making ERD an index for motor preparation that is independent of the RP (Pfurtscheller and Aranibar, 1979; Bai et al., 2006). The analyses revealed that ERD was detected in ambiguous trials, but not in the random stop signal trials from stage I (Figure 12B). Thus, at least a subset of ambiguous trials had likely already reached movement preparation and were not false alarms, but rather early cancelations.

### 7.3. Conclusion

Our findings suggest that subjects were able to cancel self-initiated movements, even after onset of the readiness potential. If a stop signal is elicited before a *point of no return* around 200 ms before movement onset, subjects are able to veto the prepared movement, while subjects cannot avoid moving when a stop signal occurs after that time point. Note, however, that the point of no return can be expected to vary from trial to trial, and that it might be impossible to determine when the point of no return has passed in single trials. This has the important implication that no critical actions should be triggered in this way, because the speed-up comes at the price of losing the opportunity to reevaluate the situation and possibly veto the action.

BCI technology offers the unique possibility of intervening in an experimental paradigm based on the momentary mental state (including intention and decision processes) of the test subject. This intriguing opportunity opens the potential for employing real-time BCIs as a research tool. While this perspective was mentioned already in the BNCI Roadmap (BNCI Horizon 2020, 2015), the presented study (Schultze-Kraft et al., 2016a) is, to the best of our knowledge, its first realization. The key point in our study is the capability of allowing for instantaneous feedback of motor intentions to subjects in real time (Blankertz et al., 2006; Salvaris and Haggard, 2014), thereby extending the important line of experimental work on the nature of predictive brain activity preceding self-initiated movements (Haggard and Eimer, 1999). This novel approach allowed us to elucidate a fundamental question in cognitive neuroscience, thereby demonstrating the potential of a Brain-Computer Interface as a powerful research tool.

## 8. Analyzing natural music listening

In this section, we show how BCI technology can be applied to the study of the processing of music. In particular, we propose a regression-based method that enables the extraction of cortical responses to note onsets in music from the continuous EEG. The extracted continuous brain responses are used to assess the brain-stimulus synchronization with a measure called Cortico-Acoustic Correlation (CACor). Several examples show the application of CACor in a range of analysis scenarios related to music perception.

### 8.1. Context: neural processes in real world experiences

Brain states during real-word experiences have attracted growing research interest in the past decade (Hasson, 2004; Hasson et al., 2010; Dmochowski et al., 2012; Hasson and Honey, 2012; Gaebler et al., 2014); Listening to music is one example of an ongoing real world experience that relies on structured auditory input that can be subject to many forms of audio signal analysis. At the same time, listening to music is one of the richest human experiences (Altenmüller and Schlaug, 2013) encompassing sensory, sensorimotor, cognitive, affective, and memory-related processes. Studying how brain dynamics underlying perceptual and cognitive processes unfold along the structure of a naturalistic music stimulus has therefore been recognized as a fruitful approach for deepening the understanding of the transformation of sensory input into human experience (Alluri et al., 2012, 2013; Sturm et al., 2014; Jäncke et al., 2015).

In the music domain, linear classification methods have, beyond measures of discriminability, provided knowledge about the neural representations of complex musical sounds (Schaefer et al., 2011; Treder et al., 2014). Unsupervised ICA-based approaches identified common features in the EEG of music listeners (Cong et al., 2012, 2013; Thompson, 2013). The results support the idea that the waveform envelope (which contains information about the timing of note onsets) is reflected in EEG and therefore provides a good starting point for linking music signals and brain signals. Likewise, in the domain of speech processing, cortical onset responses that reflect changes in the waveform envelope (termed Envelope Following Responses or EFRs), have been a target of interest for a long time (Purcell et al., 2004; Aiken and Picton, 2006, 2008).

### 8.2. Two studies investigating continuous listening experience

**Figure 13:**
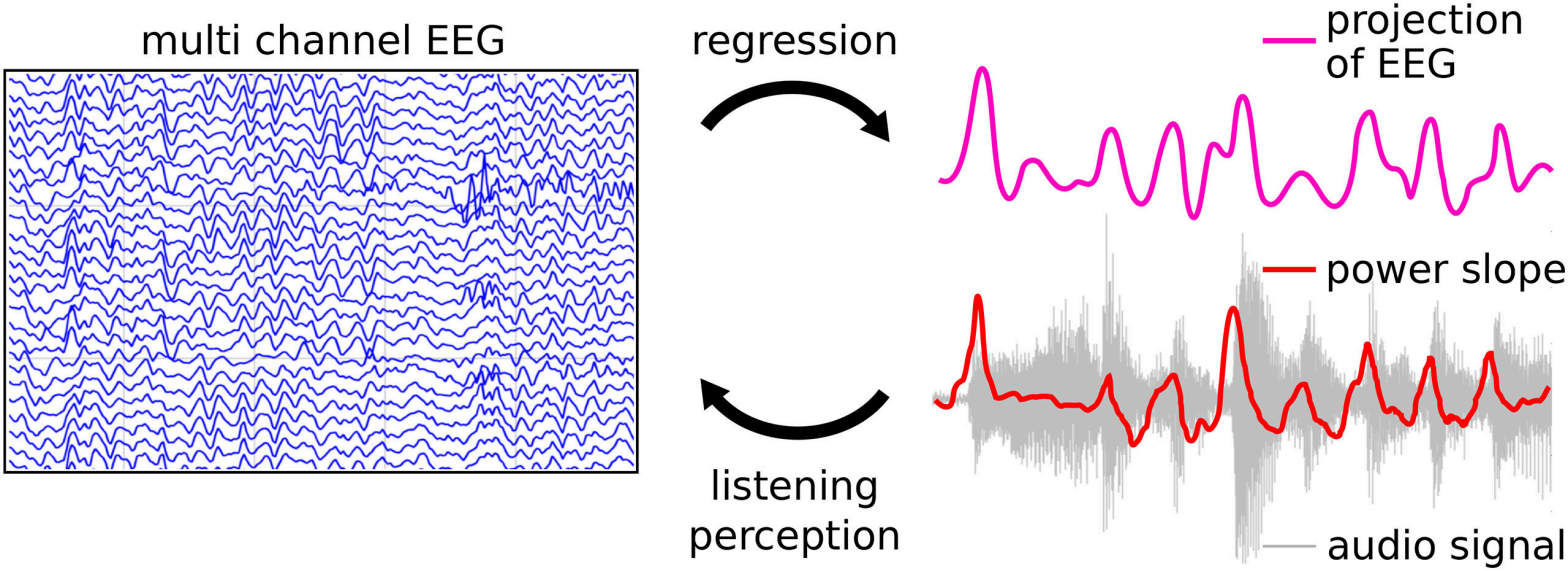
**Regression approach for extracting neural correlates of music perception**. The perception of sound is reflected in the brain signals, the power slope of the sound waves being a crucial factor. Linear Ridge Regression is applied to the temporally embedded multichannel EEG signal using the audio power slope as a target function. This results in a spatio-temporal filter (regression weight matrix) that can be applied to new data. It reduces the multichannel EEG to a one-dimensional projection that can subsequently be examined with respect to Cortico-Acoustic correlation (CACor).

**Figure 14:**
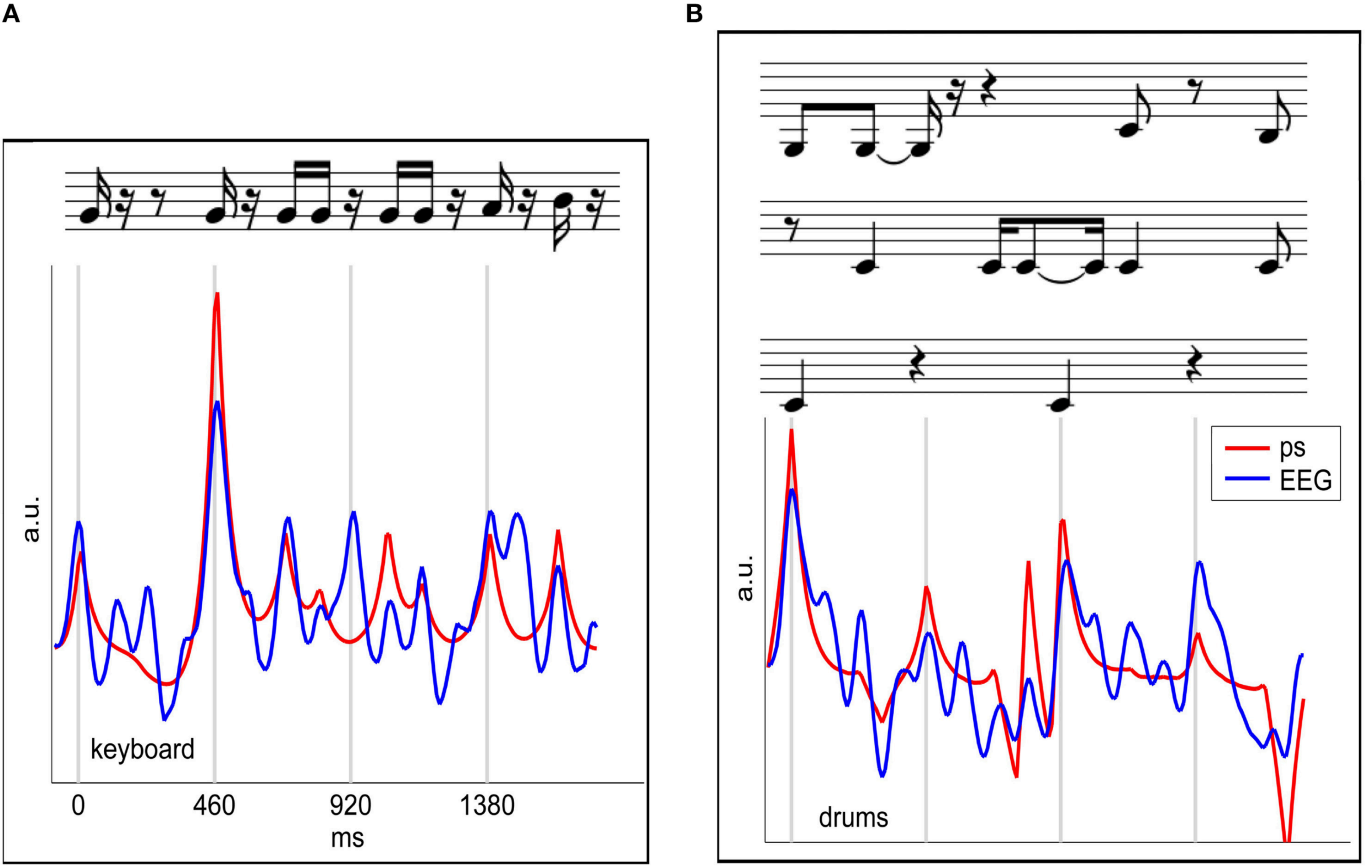
**EEG projections reflecting cortical responses to note onsets**. The two examples of keyboard **(A)** and bass **(B)** show segments of an extracted EEG projection (blue) for a single stimulus presentation and a single subject and the respective audio power slope (red). Note that in the optimization procedure a time lag between stimulus and brain response is integrated in the spatio-temporal filter, and that, consequently, the EEG projections shown here are not delayed with respect to the audio power slope. Figure adapted from Sturm et al. (2015b).

Here, we review a novel approach that utilizes the relationship of the EEG signal to the audio waveform envelope in an analysis framework that is applicable in any experimental setting in which EEG recordings and stimulus waveforms are available. As shown schematically in Figure 13 Linear Ridge Regression with the audio power slope as a target function is used to extract continuous cortical onset responses from the EEG signal of the music listener. Note that regression with the audio power *slope* capitalizes on the brain's sensitivity to change and represents a further refinement of previous EFR-related methods. Two examples of EEG projections and the respective power slope are given in Figure 14. Based on the extracted EEG projections, a measure of brain-stimulus synchronization called Cortico-Acoustic correlation (CACor) is developed (for details of the method see Sturm et al., 2015a; Sturm, 2016). We demonstrate that CACor can be applied for investigating brain-stimulus synchronization in experimental settings related to different aspects of music perception (Sturm et al., 2015b). We also provide examples of how CACor can be employed in the complementary analysis of EEG signals, behavioral measures, and audio signal analysis (Sturm et al., 2015a).

**Figure 15:**
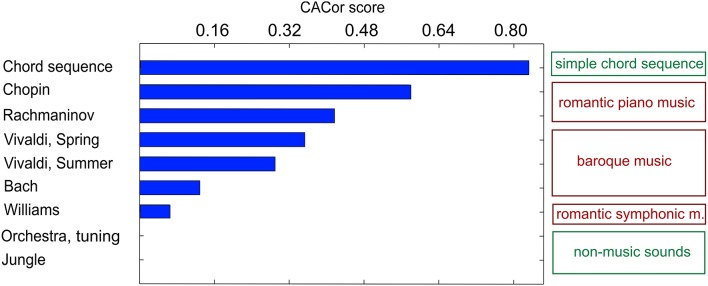
**The CACor score profile for the set of nine stimuli can be interpreted as a measure of reliability of the occurrence of significant CACor that can be compared across stimuli**. The distribution of CACor scores for the set of nine stimuli is significantly correlated (*r* = 0.9, *p* = 0.005) with the distribution of coordination scores (not shown here) that quantify how strongly coordinated behavioral responses a stimulus produces (for details on the calculation of CACor and coordination scores see Sturm et al. (2015a)). The description on the right suggests that stimuli from the same category have similar CACor measures. Figure adapted from Sturm et al. (2015a) with permission.

In a first study that explored the perception of naturalistic music, nine subjects passively listened to auditory stimuli from various sound categories, including full-length romantic piano pieces as well as simple tone sequences and natural (non-music) soundscapes (Sturm et al., 2015a). In a separate behavioral experiment, continuous ratings of the perceived tension in the same stimuli were obtained from an independent listener group. The regression approach (Figure 13) reduced the 61-channel EEG to one-time course optimally reflecting note onsets. The EEG projection was utilized to determine the Cortico-Acoustic Correlation (CACor). Significant CACor was detected in the individual listener's EEG signals of single presentations of full-length complex naturalistic music stimuli. The reliability of the occurrence of significant CACor in the group of participants differed among stimuli. It co-varied with the stimuli's average magnitudes of sharpness, spectral centroid, and rhythmic complexity. In particular, the subset of stimuli effecting a consistently strong CACor in the EEG participants (indicated by a high CACor score in Figure 15) also produced strongly coordinated tension ratings in the (independent) group of participants of the behavioral experiment. This relation between CACor and behavioral measures provides a first tentative link between neurophysiological responses to low-level acoustic events and the more cognitive-affective experience of tension in music. It is a first step toward bridging the gap between behavioral studies related to the perception of complex music and electrophysiological studies that use simplified musical stimulus material. If CACor scores are viewed as a measure of neural reliability, our findings add an interesting novel aspect to previous findings in which between-subject reliability of neural processing of naturalistic audiovisual stimuli indicates arousing and threatening passages (Dmochowski et al., 2012) and predicts the behavioral responses of large audiences (Dmochowski et al., 2014). Within this scope, our results indicate a more global, but very specific link between neural reliability and music stimulus features that might be useful for predicting listener behavior in the future.

In a second EEG study, music clips representing “rudimentary music” were presented to 11 subjects (Sturm et al., 2015b). These clips featured three instruments (keyboard, drums, and bass) playing repetitive music sound patterns, either in an ensemble version (resembling minimalistic electro-pop) or in the corresponding three solo versions (for details on stimuli and paradigm see also Treder et al., 2014). In the ensemble presentations, subjects were instructed to focus on just one of the instruments during each clip. For each instrument, a Linear Ridge Regression model was trained that extracted the EEG projection that represented the sequence of note onsets in the audio signal of the respective solo voice in an optimal way. In a second step, these instrument-specific filters were applied to EEG recorded during the ensemble presentations. CACor of the extracted EEG projections and the solo version of the music clip were assessed in order to probe whether a neural representation of the solo parts is present that is congruent to the natural ability of the subjects to perceive the single instrument's “voices” within the ensemble. Our results showed that the reflection of the melody instrument keyboard in the EEG exceeds that of the other instruments by far, suggesting a high-voice superiority effect in the neural representation of note onsets. The results further indicate that focusing attention on a particular instrument can enhance this reflection. We conclude that, in principle, the neural representation of tone onsets at the level of early auditory ERPs can parallel the perceptual segregation of ensemble music.

### 8.3. Conclusion and outlook

In summary, the machine learning based multivariate methods for EEG analysis obviates the need to present a high number of stimulus repetitions, thereby paving the avenue for studying the physiological effect of long, complex stimuli, such as full-length pieces of natural music. The approach can provide a neural representation that parallels the separate streams a listener perceives in multi-voiced music. The proposed method therefore represents a promising tool for investigating auditory stream segregation in naturalistic listening scenarios. While the investigations discussed in this section do not require the real-time capability of BCI technology, closely related research may profit from the techniques presented here. EEG-enhanced assistive listening technology aims at recognizing cortical responses to the sound envelope as a promising way to determine the attended speech stream in complex listening situations (Ding and Simon, 2012; O'Sullivan et al., 2015; Akram et al., 2016). Through this, the function of hearing aids/auditory prostheses may be adapted in a situation-dependent manner (Mirkovic et al., 2015; O'Sullivan et al., 2015).

## 9. Discussion

Most of the work on applications of BCI technology beyond communication and control is based on fundamental studies in cognitive science, psychophysics, and neuroscience. The results have been achieved in experimental studies that were confined to carefully controlled situations that limit fluctuating factors of natural tasks and behavior in order to exclude confounding variables. This is a reasonable approach for a rigorous investigation of fundamental concepts. In order to pave the way for incorporating neurotechnology into real-life applications, however, there needs to be a paradigm shift toward allowing more complex scenarios in neurocognitive studies. In this respect, it is important to note that increased noise (e.g., movement artifacts) is just one of the problems being faced.

To illustrate a more severe kind of challenge, we take the example of monitoring cognitive workload. There is a wealth of literature on the fundamental aspects of this topic, and a reliable system for real-time estimation of the current level of the user's workload would have useful applications in many areas. Studies have shown that the power of parietal alpha activity is negatively correlated with cognitive workload in visual tasks (Gevins and Smith, 2003; Holm et al., 2009). But in a complex scenario with a continuously changing visual background (such as driving a car), the alpha rhythm might be completely blocked already, such that no further decrease due to additional workload can be detected (Kohlmorgen et al., 2007). Other studies have shown that the parietal alpha amplitude *increases* with workload in non-visual tasks (Legewie et al., 1969; Galin et al., 1978; Markand, 1990), presumably as a cause of inhibition in order to focus resources to the relevant neural processes (Klimesch et al., 1999). In a natural task of high workload, the demands can change between the modalities. There might be phases when retrieving experience from memory or motor programs is critical, while in other phases the focus is on the analysis of the visual scene. The expected effect on parietal alpha is then reversed, such that this feature might be difficult to exploit in a natural task context.

A related challenge in the transfer to more complex scenarios is the sensitivity vs. specificity trade-off. Fundamental studies with confined settings have repeatedly demonstrated the high sensitivity of EEG for retrieving certain aspects of perception or cognition. Potential applications of neurotechnology often refer to this fact, e.g., when promising higher precision compared to behavioral measures. The effects found in the EEG, however, are often not very specific; the interpretation of the effect is only valid under the highly constrained experimental setting. In situations of more complex scenes or tasks there might be other factors, unrelated to the variable of interest, that can cause either the same or a reversed effect, potentially annihilating the usability of the EEG-based measure that was previously found viable in a laboratory setting.

This review excluded the aspect of a deployable setup for acquiring the brain signals. For some of the discussed applications the usual EEG hardware is appropriate (BCI as research tool, Section 7), for others a less intrusive setup is desirable (Neurousability, Section 4), while still others critically depend on the future availability of truly deployable devices (HCI applications, Section 6). Developments in this directions are the recently developed, invisible (Nikulin et al., 2010), and easy to set up, gel-free EEG electrodes (Zander et al., 2011; Guger et al., 2012; Mullen et al., 2015). Electrodes can also be placed so that they are barely visible in the ear (Looney et al., 2014; Goverdovsky et al., 2016), on the ear (Norton et al., 2015), or, as printed electrode arrays, around the ears (Debener et al., 2015). In addition, miniaturized mobile EEG systems (De Vos et al., 2014; Stopczynski et al., 2014) and NIRS systems are presently being developed (Piper et al., 2014; Von Lühmann et al., 2015). New affordable eye trackers (Dalmaijer, 2014) and the increasing commercial interest in wearable physiological sensors, such as heart-rate sensors in smart watches or a glucose sensor in a contact lens (“Google Contact Lens”), likewise promise to be beneficial for further development.

The (potential) applications of BCI technology beyond communication and control presented here have different levels of maturity. BCIs are ready for use as research tools (Sections 7 and 8). Using BCI technology to access certain user variables based on neural signals within a development cycle in order to optimize devices and interfaces is within reach for some application areas (Section 4). For other fields of applications (Sections 5 and 6), we expect that intense and challenging research is still necessary to pave the way to actual applications. The studies reviewed in this article are just the beginning (resp. already the second step) in this enterprise, which will hopefully be interesting and attractive to many researchers.

## Author contributions

BB made the concept of the review, wrote Abstract, Introduction, Overview, and Conclusion, and revised all sections. SH wrote Section 3, LA Section 4, SD Section 5, MU, MW, and BB Section 6, MS Section 7, and IS Section 8. GC contributed to the research reviewed in Sections 3–5 and 8. KM contributed to the research reviewed in Section 4. All authors read, revised, and approved the manuscript.

## Funding

This work was supported by a grant from the German Federal Ministry of Education and Research (BMBF) under contract No. 01GQ0850. Furthermore, some of the reviewed research has received funding from the European Union Seventh Framework Programme (FP7/2007-2013) under grant agreement No. 611570. SH was supported by a Marie Curie International Outgoing Fellowship (grant No. 625991) within the 7th European Community Framework Program. IS's work was supported by the Berlin School of Mind and Brain and by the Christiane-Nüsslein-Volhard Foundation. KM gratefully acknowledges funding by the Federal Ministry of Education and Research (BMBF) under the projects Adaptive BCI (FKZ 01GQ1115) and Berlin Big Data Center BBDC (01IS14013A), in part by the German Research Foundation (GRK 1589/1), and by the Brain Korea 21 Plus Program through the National Research Foundation of Korea funded by the Ministry of Education.

### Conflict of interest statement

The authors declare that the research was conducted in the absence of any commercial or financial relationships that could be construed as a potential conflict of interest.
